# Is the cholesterol-perfluoroalkyl substance association confounded by dietary fiber intake?: a Bayesian analysis of NHANES data with adjustment for measurement error in fiber intake

**DOI:** 10.1186/s12940-022-00923-2

**Published:** 2022-11-22

**Authors:** Matthew W Linakis, Paul Gustafson, Bruce C Allen, Annette M Bachand, Cynthia Van Landingham, Debra R Keast, Matthew P Longnecker

**Affiliations:** 1 Ramboll US Consulting, Inc., 3214 Charles B Root Wynd #130, Raleigh, NC 27612 USA; 2 Department of Statistics, University of British Columbia, Vancouver, Canada; 3 Independent consultant, Chapel Hill, North Carolina USA; 4 Ramboll US Consulting, Inc, Amherst, Massachusetts USA; 5 Ramboll US Consulting, Inc, Monroe, Louisiana USA; 6 Food & Nutrition Database Research, Inc, Bangor, PA USA

**Keywords:** Serum cholesterol, PFAS, Confounding, Dietary fiber, Bayesian models, Measurement error correction

## Abstract

**Background:**

Serum concentrations of total cholesterol and related lipid measures have been associated with serum concentrations of per- and polyfluoroalkyl substances (PFAS) in humans, even among those with only background-level exposure to PFAS. Fiber is known to decrease serum cholesterol and a recent report based on National Health and Nutrition Examination Survey (NHANES) showed that PFAS and fiber are inversely associated. We hypothesized that confounding by dietary fiber may account for some of the association between cholesterol and PFAS.

**Methods:**

We implemented a Bayesian correction for measurement error in estimated intake of dietary fiber to evaluate whether fiber confounds the cholesterol-PFAS association. The NHANES measure of diet, two 24-h recalls, allowed calculation of an estimate of the “true” long-term fiber intake for each subject. We fit models to the NHANES data on serum cholesterol and serum concentration of perfluorooctanoic acid (PFOA) and two other PFAS for 7,242 participants in NHANES.

**Results:**

The Bayesian model, after adjustment for soluble fiber intake, suggested a decrease in the size of the coefficient for PFOA by 6.4% compared with the fiber-unadjusted model.

**Conclusions:**

The results indicated that the association of serum cholesterol with PFAS was not substantially confounded by fiber intake.

**Supplementary Information:**

The online version contains supplementary material available at 10.1186/s12940-022-00923-2.

## Background

Per- and polyfluoroalkyl substances (PFAS) have been used in manufacturing of a variety of products, including consumer-use products such as textiles that are stain or fire resistant [1, 2]. In the general population exposure occurs mainly via contaminated food; other sources such as contaminated water are important in selected settings [3]. Most people in developed countries have detectable amounts of PFAS in their serum [3]. Epidemiological studies have suggested associations between serum concentrations of PFAS and serum concentrations of total cholesterol and related lipid measures in humans, even among those with only background-level exposures [4, 5]. Although a few animal studies have suggested an increase in serum cholesterol with increasing PFAS dose [6, 7], most show a decrease in cholesterol [5]. Nonetheless, the PFAS – cholesterol association was proposed as the point of departure in risk assessments by the European Food Safety Authority (EFSA) [8] and the California Environmental Protection Agency [9]. A recent critique of the EFSA risk assessment questioned the causality of the PFAS – cholesterol association due to uncertainties about the biologic mechanism [10]. In addition, occupational studies among workers whose PFAS levels were above background have not consistently found evidence of associations between PFAS and cholesterol [11, 12]. In an epidemiologic study by Fitz-Simon et al., marked lowering of PFAS exposure results in no change in serum cholesterol [13]. Furthermore, a large epidemiological study with an unusually high exposure to perfluorooctanoic acid (PFOA) did not find evidence of an association between PFOA and cardiovascular disease [14]. Large doses of PFOA had no effect on serum cholesterol in a clinical trial [15]. Many potential biologic mechanisms that might account for the cholesterol-PFAS association in humans have been studied in experiments [5, 16]. For example, PFAS may alter cholesterol production by hepatocytes, alter the circulation of cholesterol in plasma, or interfere with bile acid metabolism or reabsorption from the intestine and thereby affect hepatic cholesterol production. However, many challenges have arisen in the interpretation of these data because of interspecies differences in cholesterol metabolism and diet (esp. fat content), uncertainties about the relevant doses in experimental models, and inconsistent results. More data from humanized mice may help resolve the issue, though other approaches may also be useful.

**Fig. 1:**
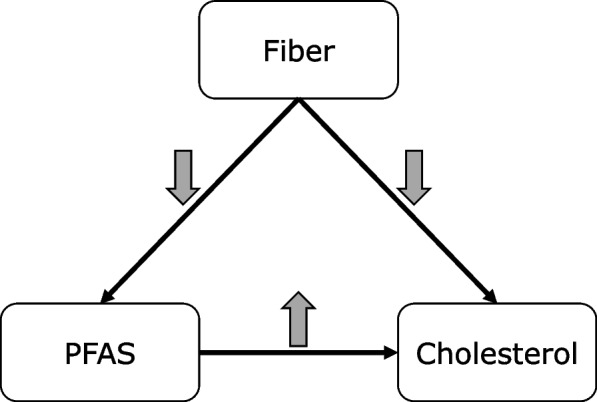
Directed acyclic graph showing relationships between fiber, PFAS, and cholesterol. Grey arrows indicate the direction of the association, specifically, fiber has an inverse relationship with PFAS concentration and cholesterol levels, while PFAS concentration has a positive correlation with cholesterol levels

A possible explanation for the contradictory epidemiologic results is confounding by dietary fiber intake [5, 16]. This explanation is plausible because an inverse relationship between dietary fiber and serum cholesterol has been suggested for many years [17], and a recent report based on National Health and Nutrition Examination Survey (NHANES) provided evidence of an inverse association between serum PFAS and fiber intake among adults aged 20 years and older [18]. A simplified directed acyclic graph depicting the potential confounding is shown in Fig. 1. Because fiber decreases both serum cholesterol and serum PFAS, not adjusting for it in epidemiologic analyses could produce the appearance of a positive relation, even if none exists.

The biologic mechanisms underlying the relationships of fiber with serum PFAS and serum cholesterol are as follows. Soluble fiber is a bile acid sequestrant [19]. Bile acid sequestrants in the intestine have two key effects. First, they cause an upregulation of bile acid synthesis in the liver, which lowers serum cholesterol [19]. Second, bile acid sequestrants have been shown to decrease serum PFAS by increasing fecal excretion, which is probably due to binding of PFAS by fiber [5, 20, 21].

We investigated the effect of confounding by fiber intake on the PFAS – cholesterol association using NHANES data. We focused on PFOA, perfluorooctane sulfonic acid (PFOS), and perfluorononanoic acid (PFNA) because these PFAS have been most consistently related to serum cholesterol in epidemiologic studies [4, 5]. Because of evidence suggesting a stronger inverse relationship between serum cholesterol and soluble dietary fiber than total dietary fiber [17], we conducted separate analyses for the two fiber variables. We recognized that due to day-to-day variation in fiber intake, error in measurement of fiber intake might result in an underestimate of the confounding it caused. To minimize such residual confounding, we implemented a Bayesian correction for measurement error [18, 22, 23]. Bayesian correction for measurement error has previously been described for an exposure of interest (e.g., [24]); in our context, however, the correction was for a confounding factor, meaning a related but slightly different approach was required, as described below.

## Methods

### Data and data manipulations

NHANES is an ongoing survey of civilian non-institutionalized people residing in the U.S., and it is conducted using a complex sampling design that oversamples selected minorities, income groups, and ages [25]. Data from NHANES are publicly available from (https://wwwn.cdc.gov/nchs/nhanes/Default.aspx). From 2003–2016, cholesterol levels were measured among NHANES participants aged 12 years and older who provided a blood sample. PFAS levels, specifically PFOA, PFOS, and PFNA, were measured in a random sample of one-third of these study subjects. All NHANES participants were asked to complete a 24-h dietary recall on two occasions 3–10 days apart [26]. The first interview took place in person and the second interview was conducted over the phone. NHANES data includes daily energy (kcal) and daily total dietary fiber (g) intake as determined by USDA using their food composition data, i.e., the U.S. Department of Agriculture (USDA) Food and Nutrient Database for Dietary Studies (FNDDS), to calculate energy (kcal) and total dietary fiber (g) intake from foods reported in NHANES dietary interviews. A food composition database developed by the Nutrition Coordinating Center (NCC), University of Minnesota, Minneapolis, MN, i.e., the NCC Food and Nutrient Database, was used to add soluble fiber data to the FNDDS, which allowed for the calculation of soluble fiber intake by NHANES respondents (Supplemental materials, S1). The fiber intake data reflect both the amount and fiber composition of foods consumed. To focus our analyses on diet composition, we estimated daily fiber intake relative to the daily caloric intake. We calculated the energy intake-adjusted fiber variables as the residuals of linear regression models of dietary fiber (g/d) on energy (kcal/d) intake [27].

We limited our study population to NHANES participants aged 20 years or older with complete serum PFAS and serum cholesterol data, and 2 days of complete dietary intake data. More than 99% of our study population had detectable concentrations of PFOA, PFOS, and PFNA in serum. For years 2013–2014 and 2015–2016, in which the linear and branched isomers of PFOA and PFOS were measured separately, we calculated the sum of these isomers. PFAS values below the limit of detection (LOD) were imputed by the CDC as LOD/√2.

To ensure that fiber intake did not act as a surrogate for other confounding variables, we extracted data for additional variables from the NHANES data set. We considered age, sex, race/ethnicity, body mass index, income to poverty ratio, smoking, dietary saturated fat, dietary cholesterol, and NHANES wave as “secondary” confounders. Additional details are provided in the Supplementary Materials, Section S2 and Figure S1.

To reduce the influence of outliers on the study results, we excluded subjects whose ln-transformed energy intake-adjusted fiber values (shifted by 40 g/d to avoid taking the log of a number ≤ 0) or ln-transformed cholesterol value fell outside the 99^th^ percentile of the normal distribution, i.e., > 2.57 standard deviations from the mean. Ln transformations were required to normalize the data. We also excluded subjects who reported using cholesterol-lowering medications (cholestyramine, colesevelam, colestipol, ezetimibe, orlistat, any statin, or Vytorin). Our analyses were based on 7,242 subjects with complete data.

### Ordinary least squares regression

As a reference point, we conducted ordinary least squares (OLS) regression with ln-transformed cholesterol as the outcome variable and ln-transformed energy intake-adjusted fiber (shifted by 40 g/d) as the confounding variable of interest, with fiber intake defined as the average over the two days. We created separate models for the three PFAS compounds and we considered a change of ≥ 10% in the coefficient for the PFAS compound after adjusting for fiber as evidence of confounding [28]. The secondary confounders were included in all models and all non-categorical confounders were centered. In sensitivity analyses, we evaluated the addition of a quadratic PFAS term with Akaike Information Criterion. Note that in OLS regression, the success of adjustment for confounding is based on the assumption that the confounder can be observed without measurement error.

### Bayesian adjustment for measurement error

Several frequentist and Bayesian methods have been suggested to correct for measurement error in model covariates [22–24, 29–31]. Bayesian analyses estimate the posterior distribution of unknown parameters based on prior information combined with information gained from available data, and they generally allow for the greatest flexibility with few assumptions.

Following an approach outlined by Bartlett and Keogh [24], we assumed that $Y$ is the response variable (cholesterol), $X$ is the exposure variable (PFOA, PFOS, or PFNA), $F$ is the confounding variable of interest (fiber intake), and ${\varvec{C}}$ represents the secondary confounding variables. If *F* could be observed, then a regression model such as

1 $$\left({\it{Y}}|\it{X,F,C}\right)\sim\text{N}\left({\beta }_{0}+{\beta }_{X}\it{X}+{\beta }_{F}F+{{{\varvec{\beta}}}_{c}}^{T}{\varvec{C}},{\sigma }^{2}\right)$$

could be fit directly, and this model contains the parameters of interest. Note that with (1) being the relationship of interest, we would include components in ***C*** that we suspect also confound the exposure-disease relationship (as we suspect of *F*).

With *F* being latent however, we can’t fit (1) alone to data. We must augment with two further models. The first of these models is (*F*|*X,C*), with a natural form being

2 $$\left({\it{F}}|\it{X, C}\right)\sim\text{N}\left({\gamma }_{0}+{\gamma }_{\it{X}}{X}+{{{\varvec{\gamma}}}_{c}}^{T}{\varvec{C}},{\omega }^{2}\right)$$

An additional component is needed to link the latent and measured values of dietary fiber. Presume that subjects have two noisy measurements, ${F}_{1}^{*}$ and ${F}_{2}^{*}$. In full generality, it is necessary to complement Eqs. (1) and (2) with a model for (${F}_{1}^{*},$ ${F}_{2}^{*}$ | *F,Y,X,C*). However, several simplifications can be made. First, a supposition that the two measurements are “pure” replicates in the sense of being conditionally independent of one another given (*F,Y,X,C*), may be justifiable. This reduces a model for the bivariate pair of measurements into two models for the univariate measurements, i.e., for (${F}_{j}^{*}$|*F,Y,X,C*), for *j* = 1,2. Second, a “nondifferential” measurement error assumption may be justifiable, whereby the measurement error is not affected by (*Y,X,C*). This then reduces the model specification task to models for (${F}_{j}^{*}$|*F*), for *j* = 1,2. Then finally it might be justifiable to posit no systematic bias in the measurement. In such a case, a specification as simple as

3 $$\left({F}_{j}^{*}|F\right)\sim {\text{N}}\left(F,{\tau }^{2}\right)$$

for *j* = 1,2, would suffice. Although subtleties of the present problem may call for a richer model, an initial evaluation as described is an important first step.

Conceptually, it is important to note that together (1), (2), and (3) prescribe a joint distribution for (${F}_{1}^{*}$,${F}_{2}^{*}$,*F,Y*|*X,C*), and therefore a joint distribution for (${F}_{1}^{*}$,${F}_{2}^{*}$,*Y*|*X,C*). Since the latter involves only observables, it provides a foundation for likelihood or Bayesian inference concerning all the unknown regression coefficients (β and γ) and variance parameters (*σ*^2^, *ω*^2^, *τ*^2^).

Operationally, Markov chain Monte Carlo software operates by sampling from the joint posterior distribution of latent variables and parameters given observed variables. Thus, the software output is simulated samples of (*F*_1:*n*,_ *β*, *γ*, *σ*^2^, *ω*^2^, *τ*^2^), where *n* is the number of subjects.

### Bayesian analyses

We used Stan statistical software (v 2.27) interfaced with R software (v 4.1.0) using the “RStan” package (v 2.21.2) [32]. We used a virtual machine with 4 virtual CPUs (Microsoft Azure Standard D4 v4) and 16 GiB of RAM. We used 4 chains with a burn-in proportion of 0.5, a maximum tree depth of 15, 2,500 iterations, and set the target average acceptance probability to 0.8. For all analyses, we used mixing and convergence diagnostics recommended by Gelman et al. [33]. We compared the parameter estimates for each PFAS between the three models, one unadjusted and two adjusted for fiber (total and soluble). We also examined the influence of using informative priors for regression coefficients in the Bayesian models (1)-(3) (not shown). The informative priors were normally distributed with parameters based on published data (Supplementary Material Sect. 3, Tables 1 and 2). We present results based on the models with informative priors because they converged more quickly than models with uninformative priors and there were no substantial differences in the results (not shown). The Stan model, R code, and data required to replicate both the OLS and Bayesian analyses have been provided in a supplemental file (Supplemental_Code.zip).

## Results

The descriptive statistics for the study variables are presented in Supplementary Material Table S1. As expected, based on the NHANES sampling design, selected minority racial and ethnic groups were overrepresented. There were more women than men because statin users (primarily males) were excluded. Other than minor differences due to the sampling design, the study population and the U.S. population were similar [18].

OLS regression results did not meaningfully depend on the NHANES sampling design and, to simplify the Bayesian analyses, we conducted unweighted analyses. Table 1 shows the estimated adjusted coefficients and their error estimates for fiber and PFOA from OLS and Bayesian models of ln-transformed cholesterol. OLS regression suggested a more pronounced association between fiber and cholesterol for soluble than for total fiber. After adjusting for measurement error in the Bayesian analyses, the coefficients for the fiber variables were more inverse (larger negative coefficient). The coefficients for PFOA were similar in the fiber-unadjusted OLS and Bayesian analyses and adjusting for fiber had a nominal effect (e.g., coefficients were 5.4% [OLS] and 6.4% [Bayesian] smaller after adjustment for soluble fiber). Error estimates for the PFOA coefficients were similar for the OLS-based and the Bayesian-based coefficients. Results for PFOS and PFNA were similar to those shown here although adjustment for fiber led to less attenuation of the coefficients (Supplemental Table S2). Results for Eq. (1) for all full models are shown in the Supplemental Materials (Tables S3-S5).

**Table 1:** Coefficients^a^ (error estimates^b^) for fiber(ln[g/d])^c^ and PFOA (ng/ml) in multivariable models^d^ of ln(serum cholesterol), according to fiber variable included in model and type of model; *n* = 7,242

Fiber variable^c^ included in model	ln-transformed energy intake-adjusted fiber (ln[g/d])		PFOA (ng/ml)	
	OLS	Bayesian	OLS	Bayesian
None	N/A	N/A	2.59 (0.75)	2.66 (0.71)
Total fiber	-1.92 (1.58)	-2.35 (1.58)	2.53 (0.75)	2.59 (0.72)
Soluble fiber	-2.90 (1.06)	-3.53 (1.49)	2.45 (0.75)	2.49 (0.72)

^a^To simplify the presentation coefficients and error estimates were multiplied by 10^–3^ for PFOA and 10^–2^ for fiber

^b^Ordinary least squares (OLS) error estimates are standard errors and Bayesian error estimates are standard deviations

^c^Modeled as ln-transformed energy intake-adjusted fiber

^d^All models were adjusted for the secondary covariates energy intake, saturated fat intake, dietary cholesterol intake, age, sex, race/ethnicity (5 categories), income to poverty ratio, smoking (4 categories, treated as an ordinal variable), and a linear and a quadratic wave variable

## Discussion

We investigated the effect of confounding by fiber intake on the association between PFAS compounds and serum cholesterol using OLS regression. Based on NHANES data, we considered total and soluble fiber and focused our analyses on diet composition. To determine if measurement error in the fiber variables affected our ability to adjust for confounding, we used Bayesian methods to correct for measurement error. We focused on the PFAS compounds PFOA, PFOS, and PFNA because previous studies have suggested that these compounds are positively associated with serum cholesterol levels [4, 34–36] and inversely associated with dietary fiber [18]. More than 99% of our study subjects had detectable concentrations of the three compounds. Although the NHANES data are based on a complex sampling design, adjusting for the variables that determined the sampling fractions (age, race/ethnicity, income) allowed us to conduct unweighted analyses [37], which simplified the Bayesian approach.

While mechanisms involving hepatic, lipoprotein, and bile acid metabolism have been proposed as explanations for observed association between PFAS and serum cholesterol in humans, a clear mechanism has not been elucidated [5, 16]. Both Andersen et al. and Fragki et al. suggested that the PFAS – cholesterol association may be explained by uncontrolled confounding by fiber intake. In our analyses of the association based on data from NHANES 2003–2016 (*n* = 7,242), adjusting for fiber had a nominal effect in both OLS regression analyses and Bayesian analyses that adjusted for measurement error.

Our study had several limitations. First, use of NHANES data meant our analyses were cross-sectional. However, the cholesterol-fiber interrelations may have been near a steady state. The three PFAS studied have half-lives on the order of years [12, 38–40] and serum cholesterol measures vary only a small amount from day-to-day [41, 42]. Another limitation was that the Bayesian analyses were based on multiple assumptions which may have been violated in our study. This included the unlikely assumption that, except for fiber, all model covariates were measured without error. We further assumed that measurement error affecting the fiber variable was unbiased despite suggestions that relying on recall can lead to biased estimates of dietary intake [43]. The Bayesian analyses on the full data set (*n* = 7,242) were computationally intensive and took an extraordinarily long time to run. They were only practical after reducing the number of iterations to 2,500 per chain and decreasing the target average acceptance probability to 0.8. Each analysis still took approximately 10 days to complete but chain convergence was acceptable. Implementing alternative approaches such as approximate Bayesian inference (e.g., the R-INLA package (r.inla.org)) could be considered in the future. Strengths of our study included our focus on diet composition by estimating fiber intake relative to total caloric intake, and the comparison of results for total and soluble fiber.

## Conclusions

Our findings suggest that adjusting the association for fiber had a nominal effect on the results and that adjusting for measurement error did not meaningfully change the results. The associations between PFAS compounds and cholesterol in serum seem unlikely to be explained by confounding by dietary fiber intake. Elucidation of the biologic mechanism accounting for the association will require laboratory experiments, possibly in humanized mice.

## Supplementary Information

**Additional file 1.**

**Additional file 2.**

**Additional file 3.**

**Additional file 4.**

## Data Availability

The NHANES and FNDDS databases are freely available and can be found at the following locations: NHANES: https://wwwn.cdc.gov/nchs/nhanes/Default.aspx FNDDS: https://www.ars.usda.gov/northeast-area/beltsville-md-bhnrc/beltsville-human-nutrition-research-center/food-surveys-research-group/docs/fndds-download-databases/ Additionally, the NCC Food and Nutrient database can be licensed from the following location: http://www.ncc.umn.edu/food-and-nutrient-database/. Because the last database is not freely available, we have also provided our database (data.csv) and our database with data dictionary (data_w_dictionary.xlsx) in the Supplemental Materials.

## Abbreviations

PFAS: Per- and polyfluoroalkyl substances
NHANES: National Health and Nutrition Examination Survey
PFOA: Perfluorooctanoic acid
EFSA: European Food Safety Authority
PFOS: Perfluorooctane sulfonic acid
PFNA: Perfluorononanoic acid
USDA: U.S. Department of Agriculture
FNDDS: Food and Nutrient Database for Dietary Studies
NCC: Nutrition Coordinating Center
LOD: Limit of Detection
OLS: Ordinary Least Squares
