# Supplementary material for: Is the cholesterol-perfluoroalkyl substance association confounded by dietary fiber intake?: a Bayesian analysis of NHANES data with adjustment for measurement error in fiber intake
Source: Environ Health. 2022 Nov 22;21:114. doi: 10.1186/s12940-022-00923-2 (PMC9682702; doi:10.1186/s12940-022-00923-2)
Supplement: Supplementary file 3 — Additional file 3. [file 12940_2022_923_MOESM3_ESM.docx]

Supplemental Material

Is the cholesterol-perfluoroalkyl substance (PFAS) association confounded by dietary fiber intake?:

a Bayesian analysis of NHANES data with adjustment for measurement error in fiber intake

Matthew W. Linakis,^a^ Paul Gustafson,^b^ Bruce C. Allen,^c^ Annette M. Bachand,^d^ Cynthia Van Landingham,^e^ Debra R. Keast,^f^ Matthew P. Longnecker^a,*^

^a^ Ramboll US Consulting, Raleigh, North Carolina, USA

^b^ Department of Statistics, University of British Columbia, Vancouver, Canada

^c^ Independent consultant, Chapel Hill, North Carolina, USA

^d^ Ramboll US Consulting, Inc., Amherst, Massachusetts, USA

^e^ Ramboll US Consulting, Inc., Monroe, Louisiana, USA

^f^ Food & Nutrition Database Research, Inc., Bangor, PA, USA

Supplement Contents

[Section S1: Soluble Fiber Methods 3](#_Toc101799757)

[Section S2: Rationale for covariates in the models 5](#_Toc101799758)

[Section S2A: Rationale for non-dietary covariates in the models 5](#_Toc101799759)

[Section S2B: Rationale for dietary variables in the models 5](#_Toc101799760)

[References 8](#_Toc101799761)

[Section S3: Informative Priors 11](#_Toc101799762)

[References 1](#_Toc101799763)3

[Supplemental Figures 14](#_Toc101799764)

[Figure S1 14](#_Toc101799765)

[Supplemental Tables 1](#_Toc101799766)6

[Table S1 1](#_Toc101799767)6

[Table S2: 1](#_Toc101799768)7

[Table S3A](#_Toc101799769) 18

[Table S3B 19](#_Toc101799770)

[Table S3C 20](#_Toc101799771)

[Table S4A 21](#_Toc101799772)

[Table S4B 22](#_Toc101799773)

[Table S4C 23](#_Toc101799774)

[Table S5A 24](#_Toc101799775)

[Table S5B 25](#_Toc101799776)

[Table S5C 26](#_Toc101799777)

# Section S1: Soluble Fiber Methods

The USDA Food and Nutrient Database for Dietary Studies (FNDDS) provides food composition data to calculate energy (Kcal) and total dietary fiber (g) intake from foods reported in NHANES dietary interviews. Soluble fiber intake from NHANES foods were determined by linking the FNDDS to the 2020 Food and Nutrient Database developed by the Nutrition Coordinating Center (NCC), University of Minnesota, Minneapolis, MN. The NCC Food and Nutrient Database contains food composition values for four dietary fiber components, including total dietary fiber, insoluble fiber, soluble fiber, and pectin. Total dietary fiber is defined as the sum of insoluble fiber and soluble fiber; insoluble fiber includes hemicellulose, cellulose and lignin; and soluble fiber includes pectin, gums, and mucilages. Analytical insoluble fiber values are mainly determined using the neutral detergent fiber method. Because fewer analytic values are available for soluble fiber, many of these values are calculated from total dietary fiber and insoluble fiber.

The 2020 NCC Food and Nutrient Database includes food composition data for 17,757 foods. To facilitate the linkage between the NCC and FNDDS databases, NCC also provided a datafile that included USDA food codes that were linked to 4,193 of the 17,757 foods. The 4,193 NCC foods in the linkage file included 1,255 NCC foods that were linked to 8-digit FNDDS food codes, and 2,938 NCC foods that were linked to the USDA Standard Reference (SR) Nutrient Database. The 5-digit SR food codes are the “ingredient” food codes used in the FNDDS SR-link “recipe” file to calculate the nutrient content of FNDDS foods. Because many of the FNDDS and SR codes listed in the NCC linkage file were used multiple times, there were only 989 FNDDS food codes and 1,134 SR food codes linked to the 4,193 NCC foods in the linkage file.

There were 8,940 FNDDS foods reported at least once on either day 1 or day 2 of the dietary interviews conducted in NHANES, 2003-2016. These 8,940 FNDDS foods included 6,994 fiber-containing foods, and 704 of these 6,994 foods matched one of the 989 FNDDS food codes used in the NCC linkage file. If multiple NCC food codes were matched to a FNDDS food, the NCC food descriptions were reviewed to identify the NCC food that best matched the FNDDS food. After matching NCC foods to 704 of the 6,994 FNDDS foods, there were 6,290 FNDDS foods which contain fiber that remained unmatched.

The insoluble fiber content of the 6,290 unmatched FNDDS foods were calculated using the FNDDS SR-link “recipe” database. Because SR “ingredient” codes are used multiple times in the FNDDS “recipe” file, only 2,439 different SR codes were used to calculate the fiber content of the 6,290 FNDDS foods. These 2,439 SR foods included 1,971 fiber-containing foods, and 893 of these 1,971 foods matched one of the 1,134 SR food codes used in the NCC linkage file. If multiple NCC food codes were matched to a SR food, the NCC food descriptions were reviewed to identify the NCC food that best matched the SR food. After matching NCC foods to 893 of the 1,971 SR foods using the NCC linkage file, there were 1,078 SR foods which contain fiber that remained unmatched. Each of the remaining 1,078 SR foods were assigned one of the 17,757 NCC food codes by identifying the NCC food description that best matched the SR food description. Once the SR ingredient database was complete, recipe calculations were performed to determine the insoluble fiber content of the food.

Because there were seven FNDDS releases for each of the seven waves of the combined NHANES, 2003-2016, a separate insoluble fiber database was compiled for each FNDDS release. Each FNDDS release included 4,573-5,714 foods reported at least once in any dietary interview conducted during the respective survey years, and of the 3,572-4,517 FNDDS foods that contained fiber, 388-462 matched one of the 989 FNDDS food codes used in the NCC linkage file, while 3,184-4,055 remained unmatched. After completing the SR ingredient database and performing recipe calculations for unmatched foods, the NCC fiber components were then added to each of the seven FNDDS releases. To calibrate the NCC fiber composition to the USDA total dietary fiber content, the NCC fiber component composition was expressed as a percentage of the NCC total dietary fiber content, and these percentages were then applied to the USDA total dietary fiber content. Then insoluble fiber intake from each food reported in the dietary interview was determined as the product of 100 times the insoluble fiber content (g/100 gram) times the weight (g) of food consumed, and daily insoluble fiber intake was determined by summing insoluble fiber intake from all foods reported that day.

# Section S2: Rationale for covariates in the models

## Section S2A: Rationale for non-dietary covariates in the models

We identified non-dietary variables that were determinants of both serum cholesterol concentration and serum PFAS concentration.

In the case of serum cholesterol, in previous large epidemiologic studies, the following predictors have been reported: age, sex, race/ethnicity, body mass index (BMI), education, smoking, and year (see Table 1 for Section S1, below). Using the NHANES data in our analysis (see main paper), we examined whether these factors were predictors of serum cholesterol. Age, sex, race/ethnicity, BMI, smoking, and year predicted serum cholesterol; education did not seem to be important, but when we replaced education with income to poverty ratio, a relation was clear, but in the opposite of the expected direction (higher cholesterol with higher income). We expected males would have a higher serum cholesterol than females, but the opposite was true. Males are more likely than females to be treated for hypercholesterolemia (Zhao et al., 2020). The relation of the non-dietary covariates to serum cholesterol concentration in our analysis of NHANES data is shown in Table 2 for Section S1.

Table 1. References showing data supporting a relation of a given non-dietary variable to serum cholesterol concentration

| Variable | Reference |
| --- | --- |
| Age | (Appleby et al., 1995; Bolton-Smith et al., 1991; Gardner et al., 2000; Pincherle, 1971) |
| Sex | (Gardner et al., 2000; Pincherle, 1971) |
| Race/ethnicity | (Gardner et al., 2000) |
| BMI | (Bolton-Smith et al., 1991; Pincherle, 1971) |
| Education | (Bolton-Smith et al., 1991; Gardner et al., 2000) |
| Smoking | (Appleby et al., 1995; Bolton-Smith et al., 1991; Pincherle, 1971) |
| Year | (Farzadfar et al., 2011) |

For non-dietary determinants of serum PFAS concentration, we relied on (Dzierlenga et al., 2021), because they developed a comprehensive model of serum PFAS for largely the same group of NHANES subjects as were included in the present study. Age, sex, race/ethnicity, BMI, Smoking (PFOS), year, and income to poverty ratio were important predictors (see Table 2 for Section S1 below, which shows the parameter estimates from the models fit by Dzierlenga et al). Thus, the same set of non-dietary factors were predictors in models of serum cholesterol and serum PFAS, and therefore we considered these to be confounding factors that we would adjust for when evaluating confounding of the cholesterol-fiber (or cholesterol-diet) relationship in the NHANES data.

## Section S2B: Rationale for dietary variables in the models

We identified dietary variables that were determinants of both serum cholesterol concentration and serum PFAS concentration.

In the case of serum cholesterol, experimental data in humans have shown that intake of saturated fatty acids and cholesterol increase levels (Hegsted et al., 1993). These same experimental studies show that polyunsaturated fatty acids in diet decrease serum cholesterol, but we did not further consider polyunsaturated fatty acids for inclusion in our models. Most polyunsaturated fatty acids in the diet come from sources other than fish (Kris-Etherton et al., 2000), such as cooking oil, which is not a source of dietary PFAS. Observational data on dietary fatty acids and dietary cholesterol and serum cholesterol were reviewed by (Caggiula and Mustad, 1997), who concluded that “… intakes of saturated fatty acids and dietary cholesterol are generally positively correlated with blood cholesterol in men and women.“ (Mente et al., 2017) recently confirmed these associations in a large multi-center, international cohort study.

The association of dietary fiber with decreased serum cholesterol is well-established, and has been reported in experimental studies of humans (Brown et al., 1999), and observational studies (Appleby et al., 1995; Bolton-Smith et al., 1991; Wu et al., 2007).

Energy intake is important to include in models of serum cholesterol for two reasons. First, whenever the relation of serum cholesterol to a dietary variable is examined, the analysts condition on energy intake so that the coefficients for components of diet represent diet quality rather than diet quantity (Willett et al., 1997). Second, energy intake per se has been found to be inversely related to serum cholesterol directly in observational studies (Sonnenberg et al., 1992), and studies of physical activity interventions are consistent with this, where more physical activity reduces total serum cholesterol (Mann et al., 2014).

Dietary phytosterols are known to decrease serum cholesterol (Dumolt and Rideout, 2017; Klingberg et al., 2008), but in the NHANES no data are available on dietary intake of phytosterols. If intake of phytosterols is correlated with that of dietary fiber (Klingberg et al., 2008; Li et al., 2018), then we may overestimate the inverse association of dietary fiber with serum cholesterol.

In the case of serum PFAS, the intake of foods in certain groups such as meat, dairy, eggs, and fish, have been reported by others to be related to higher serum PFAS (Averina et al., 2018; Brantsæter et al., 2013; Domingo et al., 2012; Ericson et al., 2008; Eriksen et al., 2011; Haug et al., 2010; Jain, 2014; Liu et al., 2017; Noorlander et al., 2011; Park et al., 2019; Skuladottir et al., 2015; Tittlemier et al., 2007; Vestergren and Cousins, 2013; Zhou et al., 2019). In the Dzierlenga et al. analysis, however, meat, dairy, and eggs, which would be sources of saturated fatty acids or cholesterol, were not statistically significantly related to increased serum PFAS (Table 2 for Section S1). Fish intake was associated with higher PFAS, but fish intake is not a determinant of serum cholesterol (Alhassan et al., 2017). The intake of dietary fiber and either saturated fatty acids or dietary cholesterol may be inversely related, and to show that any effect of fiber on cholesterol is in fact due to fiber and not to other factors that may influence serum cholesterol, we include saturated fatty acids and dietary cholesterol in the models.

**Table 2. Coefficients from models of ln(PFAS), from the full model used in Dzierlenga et al. (2021)**

| **Variable** | **Description** | **Units** | **β (95% CI)** | | |
| --- | --- | --- | --- | --- | --- |
|  |  |  | **PFOA (ln(ng/mL))** | **PFOS (ln(ng/mL))** | **PFNA (ln(ng/mL))** |
| (Intercept) | NA | Unitless | 1.21e+00 (9.96e-01, 1.43e+00) | 2.52e+00 (2.31e+00, 2.72e+00) | -2.39e-02 (-2.24e-01, 1.76e-01) |
| eaf_iqr | interquartile shift in energy-adjusted dietary fiber | g fiber/interquartile distance | -3.88e-02 (-6.48e-02, -1.27e-02) | -6.97e-02 (-1.01e-01, -3.87e-02) | -1.25e-01 (-1.84e-01, -6.57e-02) |
| eaf2_iqr | interquartile shift in energy-adjusted dietary fiber squared | (g fiber/interquartile distance)^2^ | NA | NA | 4.05e-02 (1.85e-03, 7.92e-02) |
| kcal_energy | energy intake | kcal energy | 7.58e-07 (-3.24e-05, 3.39e-05) | -4.10e-05 (-7.74e-05, -4.63e-06) | -5.57e-05 (-8.91e-05, -2.22e-05) |
| g_meat | meat consumption | g meat | 1.12e-04 (-7.64e-05, 3.00e-04) | 1.40e-04 (-2.16e-05, 3.01e-04) | 2.54e-04 (1.16e-04, 3.92e-04) |
| g_dairy | dairy consumption | g dairy | -1.75e-04 (-2.67e-04, -8.23e-05) | -1.91e-04 (-3.11e-04, -7.07e-05) | -1.41e-04 (-2.56e-04, -2.50e-05) |
| g_eggs | egg consumption | g eggs | 9.82e-05 (-2.20e-04, 4.16e-04) | -1.43e-04 (-5.27e-04, 2.41e-04) | 3.41e-04 (-2.32e-05, 7.04e-04) |
| g_popcorn | popcorn consumption | g popcorn | 2.45e-03 (6.75e-04, 4.23e-03) | 2.87e-03 (1.04e-03, 4.70e-03) | 2.31e-03 (3.66e-04, 4.26e-03) |
| fish_freq | Fish/shellfish consumption | Number of seafood meals last 30 days | 4.71e-03 (2.12e-03, 7.30e-03) | 8.62e-03 (5.58e-03, 1.17e-02) | 1.32e-02 (9.78e-03, 1.67e-02) |
| ridageyr | Age | Year | 6.67e-03 (5.19e-03, 8.15e-03) | 1.33e-02 (1.15e-02, 1.51e-02) | 6.70e-03 (4.84e-03, 8.56e-03) |
| bmxbmi | BMI | kg/m^2 | -5.46e-03 (-8.60e-03, -2.31e-03) | -8.64e-03 (-1.17e-02, -5.54e-03) | -5.41e-03 (-8.78e-03, -2.04e-03) |
| indfmpir | Income to poverty level | Unitless | 3.72e-02 (2.44e-02, 5.01e-02) | 3.10e-02 (1.54e-02, 4.66e-02) | 5.09e-02 (3.59e-02, 6.58e-02) |
| par_cat1 | Parity | 1 Childbirth | -1.00e-01 (-1.86e-01, -1.39e-02) | -3.30e-02 (-1.28e-01, 6.16e-02) | 4.78e-02 (-5.18e-02, 1.47e-01) |
| par_cat2 | Parity | 2 or more Childbirths | -1.42e-01 (-2.05e-01, -7.83e-02) | -1.04e-01 (-1.86e-01, -2.30e-02) | 1.36e-03 (-6.75e-02, 7.02e-02) |
| sex_cat1 | Sex | Male | 1.76e-01 (1.07e-01, 2.44e-01) | 4.14e-01 (3.34e-01, 4.94e-01) | 1.73e-01 (1.03e-01, 2.42e-01) |
| wave | NHANES wave | Wave index (05-06:0, 07-08:1…) | -1.45e-01 (-2.24e-01, -6.56e-02) | -3.63e-01 (-4.30e-01, -2.95e-01) | 9.43e-02 (-2.10e-02, 2.10e-01) |
| I(wave^2) | NHANES wave squared | (Wave index)^2^ | -1.14e-02 (-2.53e-02, 2.62e-03) | 1.84e-02 (5.06e-03, 3.17e-02) | -4.89e-02 (-6.88e-02, -2.89e-02) |
| smq_cat1 | Smoking | Former Smoker | -4.93e-03 (-4.95e-02, 3.96e-02) | -8.39e-02 (-1.29e-01, -3.83e-02) | -3.79e-02 (-9.34e-02, 1.76e-02) |
| smq_cat2 | Smoking | Smoker | -3.24e-02 (-9.12e-02, 2.64e-02) | -1.92e-01 (-2.63e-01, -1.21e-01) | -7.19e-02 (-1.25e-01, -1.88e-02) |
| smq_cat3 | Smoking | Heavy Smoker | 2.81e-02 (-5.70e-02, 1.13e-01) | -1.74e-01 (-2.51e-01, -9.74e-02) | -6.90e-02 (-1.55e-01, 1.75e-02) |
| alq_cat1 | Alcohol Use | Former Drinker | -1.26e-02 (-9.49e-02, 6.98e-02) | -3.05e-03 (-1.00e-01, 9.42e-02) | 6.36e-03 (-8.67e-02, 9.94e-02) |
| alq_cat2 | Alcohol Use | Light Drinker | 1.10e-03 (-6.86e-02, 7.08e-02) | 1.02e-02 (-7.06e-02, 9.11e-02) | 6.86e-03 (-7.52e-02, 8.89e-02) |
| alq_cat3 | Alcohol Use | Drinker | 3.72e-02 (-3.34e-02, 1.08e-01) | 3.65e-02 (-5.61e-02, 1.29e-01) | 6.20e-02 (-1.67e-02, 1.41e-01) |
| alq_cat4 | Alcohol Use | Heavy Drinker | 3.65e-02 (-4.71e-02, 1.20e-01) | 9.60e-03 (-9.60e-02, 1.15e-01) | 8.48e-02 (-1.41e-02, 1.84e-01) |
| reth_cat1 | Race/Ethnicity | Mexican American | -2.24e-01 (-3.05e-01, -1.44e-01) | -1.89e-01 (-2.61e-01, -1.16e-01) | -4.10e-02 (-1.30e-01, 4.76e-02) |
| reth_cat2 | Race/Ethnicity | Other Hispanic | -1.26e-01 (-2.11e-01, -4.22e-02) | -2.03e-01 (-3.02e-01, -1.04e-01) | 6.87e-02 (-3.29e-02, 1.70e-01) |
| reth_cat3 | Race/Ethnicity | Non-Hispanic Black | -1.47e-01 (-2.09e-01, -8.56e-02) | 1.51e-01 (7.78e-02, 2.24e-01) | 1.38e-01 (6.92e-02, 2.07e-01) |
| reth_cat4 | Race/Ethnicity | Other Race or Mixed Race | -1.09e-01 (-1.81e-01, -3.75e-02) | -2.61e-02 (-1.16e-01, 6.39e-02) | 8.32e-02 (1.54e-03, 1.65e-01) |
| edu_cat1 | Education | Some High School | 6.07e-02 (-3.39e-02, 1.55e-01) | 3.26e-03 (-9.90e-02, 1.06e-01) | -3.98e-02 (-1.17e-01, 3.72e-02) |
| edu_cat2 | Education | High School Diploma | 7.54e-02 (-9.05e-03, 1.60e-01) | 2.86e-02 (-6.47e-02, 1.22e-01) | -1.45e-02 (-8.92e-02, 6.02e-02) |
| edu_cat3 | Education | Some College of AA degree | 4.79e-02 (-4.53e-02, 1.41e-01) | 7.80e-03 (-8.81e-02, 1.04e-01) | -8.84e-02 (-1.77e-01, 6.49e-05) |
| edu_cat4 | Education | College Degree | -1.50e-03 (-8.79e-02, 8.49e-02) | -7.20e-02 (-1.72e-01, 2.83e-02) | -1.08e-01 (-1.96e-01, -2.02e-02) |

## References

Alhassan, A., Young, J., Lean, M.E.J., Lara, J., 2017. Consumption of fish and vascular risk factors: A systematic review and meta-analysis of intervention studies. Atherosclerosis 266, 87–94. https://doi.org/10.1016/j.atherosclerosis.2017.09.028

Appleby, P.N., Thorogood, M., McPherson, K., Mann, J.L., 1995. Associations between plasma lipid concentrations and dietary, lifestyle and physical factors in the Oxford Vegetarian Study. J. Hum. Nutr. Diet. 8, 305–314. https://doi.org/10.1111/j.1365-277X.1995.tb00324.x

Averina, M., Brox, J., Huber, S., Furberg, A.-S., 2018. Perfluoroalkyl substances in adolescents in northern Norway: Lifestyle and dietary predictors. The Tromsø study, Fit Futures 1. Environ. Int. 114, 123–130. https://doi.org/10.1016/j.envint.2018.02.031

Bolton-Smith, C., Woodward, M., Smith, W.C., Tunstall-Pedoe, H., 1991. Dietary and non-dietary predictors of serum total and HDL-cholesterol in men and women: results from the Scottish Heart Health Study. Int. J. Epidemiol. 20, 95–104. https://doi.org/10.1093/ije/20.1.95

Brantsæter, A.L., Whitworth, K.W., Ydersbond, T.A., Haug, L.S., Haugen, M., Knutsen, H.K., Thomsen, C., Meltzer, H.M., Becher, G., Sabaredzovic, A., Hoppin, J.A., Eggesbø, M., Longnecker, M.P., 2013. Determinants of plasma concentrations of perfluoroalkyl substances in pregnant Norwegian women. Environ. Int. 54, 74–84. https://doi.org/10.1016/j.envint.2012.12.014

Brown, L., Rosner, B., Willett, W.W., Sacks, F.M., 1999. Cholesterol-lowering effects of dietary fiber: a meta-analysis. Am. J. Clin. Nutr. 69, 30–42. https://doi.org/10.1093/ajcn/69.1.30

Caggiula, A.W., Mustad, V.A., 1997. Effects of dietary fat and fatty acids on coronary artery disease risk and total and lipoprotein cholesterol concentrations: epidemiologic studies. Am. J. Clin. Nutr. 65, 1597S-1610S. https://doi.org/10.1093/ajcn/65.5.1597S

Domingo, J.L., Jogsten, I.E., Eriksson, U., Martorell, I., Perelló, G., Nadal, M., Bavel, B. van, 2012. Human dietary exposure to perfluoroalkyl substances in Catalonia, Spain. Temporal trend. Food Chem. 135, 1575–1582. https://doi.org/10.1016/j.foodchem.2012.06.054

Dumolt, J.H., Rideout, T.C., 2017. The Lipid-lowering Effects and Associated Mechanisms of Dietary Phytosterol Supplementation. Curr. Pharm. Des. 23, 5077–5085. https://doi.org/10.2174/1381612823666170725142337

Dzierlenga, M.W., Keast, D.R., Longnecker, M.P., 2021. The concentration of several perfluoroalkyl acids in serum appears to be reduced by dietary fiber. Environ. Int. 146, 106292. https://doi.org/10.1016/j.envint.2020.106292

Ericson, I., Martí-Cid, R., Nadal, M., Van Bavel, B., Lindström, G., Domingo, J.L., 2008. Human exposure to perfluorinated chemicals through the diet: intake of perfluorinated compounds in foods from the Catalan (Spain) market. J. Agric. Food Chem. 56, 1787–1794. https://doi.org/10.1021/jf0732408

Eriksen, K.T., Sørensen, M., McLaughlin, J.K., Tjønneland, A., Overvad, K., Raaschou-Nielsen, O., 2011. Determinants of plasma PFOA and PFOS levels among 652 Danish men. Environ. Sci. Technol. 45, 8137–8143. https://doi.org/10.1021/es100626h

Farzadfar, F., Finucane, M.M., Danaei, G., Pelizzari, P.M., Cowan, M.J., Paciorek, C.J., Singh, G.M., Lin, J.K., Stevens, G.A., Riley, L.M., Ezzati, M., Global Burden of Metabolic Risk Factors of Chronic Diseases Collaborating Group (Cholesterol), 2011. National, regional, and global trends in serum total cholesterol since 1980: systematic analysis of health examination surveys and epidemiological studies with 321 country-years and 3·0 million participants. Lancet Lond. Engl. 377, 578–586. https://doi.org/10.1016/S0140-6736(10)62038-7

Gardner, C.D., Winkleby, M.A., Fortmann, S.P., 2000. Population frequency distribution of non-high-density lipoprotein cholesterol (Third National Health and Nutrition Examination Survey [NHANES III], 1988-1994). Am. J. Cardiol. 86, 299–304. https://doi.org/10.1016/s0002-9149(00)00918-8

Haug, L.S., Salihovic, S., Jogsten, I.E., Thomsen, C., van Bavel, B., Lindström, G., Becher, G., 2010. Levels in food and beverages and daily intake of perfluorinated compounds in Norway. Chemosphere 80, 1137–1143. https://doi.org/10.1016/j.chemosphere.2010.06.023

Hegsted, D.M., Ausman, L.M., Johnson, J.A., Dallal, G.E., 1993. Dietary fat and serum lipids: an evaluation of the experimental data. Am. J. Clin. Nutr. 57, 875–883. https://doi.org/10.1093/ajcn/57.6.875

Jain, R.B., 2014. Contribution of diet and other factors to the levels of selected polyfluorinated compounds: data from NHANES 2003-2008. Int. J. Hyg. Environ. Health 217, 52–61. https://doi.org/10.1016/j.ijheh.2013.03.008

Klingberg, S., Ellegård, L., Johansson, I., Hallmans, G., Weinehall, L., Andersson, H., Winkvist, A., 2008. Inverse relation between dietary intake of naturally occurring plant sterols and serum cholesterol in northern Sweden. Am. J. Clin. Nutr. 87, 993–1001. https://doi.org/10.1093/ajcn/87.4.993

Kris-Etherton, P.M., Taylor, D.S., Yu-Poth, S., Huth, P., Moriarty, K., Fishell, V., Hargrove, R.L., Zhao, G., Etherton, T.D., 2000. Polyunsaturated fatty acids in the food chain in the United States. Am. J. Clin. Nutr. 71, 179S–88S. https://doi.org/10.1093/ajcn/71.1.179S

Li, Y.-C., Li, C.-L., Li, R., Chen, Y., Zhang, M., Guo, P.-P., Shi, D., Ji, X.-N., Feng, R.-N., Sun, C.-H., 2018. Associations of dietary phytosterols with blood lipid profiles and prevalence of obesity in Chinese adults, a cross-sectional study. Lipids Health Dis. 17, 54. https://doi.org/10.1186/s12944-018-0703-y

Liu, Y., Su, J., van Dam, R.M., Prem, K., Hoong, J.Y.S., Zou, L., Lu, Y., Ong, C.N., 2017. Dietary predictors and plasma concentrations of perfluorinated alkyl acids in a Singapore population. Chemosphere 171, 617–624. https://doi.org/10.1016/j.chemosphere.2016.12.107

Mann, S., Beedie, C., Jimenez, A., 2014. Differential effects of aerobic exercise, resistance training and combined exercise modalities on cholesterol and the lipid profile: review, synthesis and recommendations. Sports Med. Auckl. NZ 44, 211–221. https://doi.org/10.1007/s40279-013-0110-5

Mente, A., Dehghan, M., Rangarajan, S., McQueen, M., Dagenais, G., Wielgosz, A., Lear, S., Li, W., Chen, H., Yi, S., Wang, Y., Diaz, R., Avezum, A., Lopez-Jaramillo, P., Seron, P., Kumar, R., Gupta, R., Mohan, V., Swaminathan, S., Kutty, R., Zatonska, K., Iqbal, R., Yusuf, R., Mohammadifard, N., Khatib, R., Nasir, N.M., Ismail, N., Oguz, A., Rosengren, A., Yusufali, A., Wentzel-Viljoen, E., Puoane, T., Chifamba, J., Teo, K., Anand, S.S., Yusuf, S., Prospective Urban Rural Epidemiology (PURE) study investigators, 2017. Association of dietary nutrients with blood lipids and blood pressure in 18 countries: a cross-sectional analysis from the PURE study. Lancet Diabetes Endocrinol. 5, 774–787. https://doi.org/10.1016/S2213-8587(17)30283-8

Noorlander, C.W., van Leeuwen, S.P.J., Te Biesebeek, J.D., Mengelers, M.J.B., Zeilmaker, M.J., 2011. Levels of perfluorinated compounds in food and dietary intake of PFOS and PFOA in the Netherlands. J. Agric. Food Chem. 59, 7496–7505. https://doi.org/10.1021/jf104943p

Park, S.K., Peng, Q., Ding, N., Mukherjee, B., Harlow, S.D., 2019. Determinants of per- and polyfluoroalkyl substances (PFAS) in midlife women: Evidence of racial/ethnic and geographic differences in PFAS exposure. Environ. Res. 175, 186–199. https://doi.org/10.1016/j.envres.2019.05.028

Pincherle, G., 1971. Factors affecting the mean serum cholesterol. J. Chronic Dis. 24, 289–297. https://doi.org/10.1016/0021-9681(71)90119-6

Skuladottir, M., Ramel, A., Rytter, D., Haug, L.S., Sabaredzovic, A., Bech, B.H., Henriksen, T.B., Olsen, S.F., Halldorsson, T.I., 2015. Examining confounding by diet in the association between perfluoroalkyl acids and serum cholesterol in pregnancy. Environ. Res. 143, 33–38. https://doi.org/10.1016/j.envres.2015.09.001

Sonnenberg, L.M., Posner, B.M., Belanger, A.J., Cupples, L.A., D’Agostino, R.B., 1992. Dietary predictors of serum cholesterol in men: the Framingham cohort population. J. Clin. Epidemiol. 45, 413–418. https://doi.org/10.1016/0895-4356(92)90042-l

Tittlemier, S.A., Pepper, K., Seymour, C., Moisey, J., Bronson, R., Cao, X.-L., Dabeka, R.W., 2007. Dietary exposure of Canadians to perfluorinated carboxylates and perfluorooctane sulfonate via consumption of meat, fish, fast foods, and food items prepared in their packaging. J. Agric. Food Chem. 55, 3203–3210. https://doi.org/10.1021/jf0634045

Vestergren, R., Cousins, I.T., 2013. 12 - Human dietary exposure to per- and poly-fluoroalkyl substances (PFASs), in: Rose, M., Fernandes, A. (Eds.), Persistent Organic Pollutants and Toxic Metals in Foods, Woodhead Publishing Series in Food Science, Technology and Nutrition. Woodhead Publishing, pp. 279–307. https://doi.org/10.1533/9780857098917.2.279

Willett, W.C., Howe, G.R., Kushi, L.H., 1997. Adjustment for total energy intake in epidemiologic studies. Am. J. Clin. Nutr. 65, 1220S-1228S; discussion 1229S-1231S. https://doi.org/10.1093/ajcn/65.4.1220S

Wu, K., Bowman, R., Welch, A.A., Luben, R.N., Wareham, N., Khaw, K.-T., Bingham, S.A., 2007. Apolipoprotein E polymorphisms, dietary fat and fibre, and serum lipids: the EPIC Norfolk study. Eur. Heart J. 28, 2930–2936. https://doi.org/10.1093/eurheartj/ehm482

Zhao, M., Woodward, M., Vaartjes, I., Millett, E.R.C., Klipstein-Grobusch, K., Hyun, K., Carcel, C., Peters, S.A.E., 2020. Sex Differences in Cardiovascular Medication Prescription in Primary Care: A Systematic Review and Meta-Analysis. J. Am. Heart Assoc. 9, e014742. https://doi.org/10.1161/JAHA.119.014742

Zhou, W., Zhao, S., Tong, C., Chen, L., Yu, X., Yuan, T., Aimuzi, R., Luo, F., Tian, Y., Zhang, J., Shanghai Birth Cohort study, 2019. Dietary intake, drinking water ingestion and plasma perfluoroalkyl substances concentration in reproductive aged Chinese women. Environ. Int. 127, 487–494. https://doi.org/10.1016/j.envint.2019.03.075

# Section S3: Informative Priors

Table 1. Informative priors for beta coefficients in models of ln(serum cholesterol). All informative priors had normal distributions with mean β and standard deviation σ(β).

| Variable | β | σ(β) | References |
| --- | --- | --- | --- |
| Intercept | 5.394 | 0.1 | (Schneider et al., 1987) (mean) |
| PFAS | - | - |  |
| Age | 0.003 | 0.0015000 | (Sonnenberg et al., 1992) |
| Sex | 0.068 | 0.0510000 | (Seidell et al., 1991) |
| Race/Ethnicity | 0 | 0.0700000 | (Frank et al., 2014) |
| IPR | 0 | 0.0180000 | (Luepker et al., 1993) (see below) |
| BMI | 0.009 | 0.0067500 | (Bolton-Smith et al., 1991) |
| Smoking | 0.018 | 0.0060000 | (Keto et al., 2016) |
| SFA | 0.001 | 0.0007500 | (Bolton-Smith et al., 1991) |
| Dietary cholesterol | 0.00007 | 0.0000525 | (Bolton-Smith et al., 1991) |
| Energy intake | -0.00002 | 0.0000150 | (Bolton-Smith et al., 1991) |
| Wave | 0 | 1 | (Carroll, 2020) |
| Wave^2^ | 0 | 1 |  |
| Fiber | - | - |  |

We used income to poverty ratio (IPR) instead of education because it had fewer missing data.

Luepker et al. 1993 (Table 2) was used as the source of information. We made the approximation that two IPR units corresponded to the low and high income categories that Luepker made, based on the relation of education to IPR in NHANES data. As income went from low to high, cholesterol generally increased, but for females in 1985-1987, it decreased. The maximum increase was males 1985-1987, where the increase was 3.5% per two IPR units, or 0.018 per IPR unit. Because of the uncertainty about the direction of effect, we set the a priori β = 0, and let 0.018 be the standard error.

Table 2. Informative priors for gamma coefficients in models of ln(energy-adjusted fiber). All informative priors had normal distributions with mean γ and standard deviation σ(γ).

| Variable | γ | σ(γ) | References |
| --- | --- | --- | --- |
| Intercept | 0 | 0.1290000 | - |
| PFAS | -0.00753 | 0.0056475 | (Dzierlenga et al., 2021) (see note below) |
| Age | 0.01 | 0.0075000 | (Lanza et al., 1987) (their Figure 2) |
| Sex | 0.2 | 0.1500000 | (Lanza et al., 1987) (their Figure 2a) |
| Race/Ethnicity | 0 | 0.2200000 | (Lanza et al., 1987) (their p 793 & Figure 1) |
| IPR | 0.08 | 0.0600000 | (Lanza et al., 1987) (their Figure 2) |
| BMI | 0 | 0.0900000 | (Fuchs et al., 1999; Platz et al., 1997) |
| Smoking | -0.31 | 0.2325000 | (Subar et al., 1990) |
| SFA | -0.1 | 0.0750000 | (Liu et al., 2002) (their Table 1) |
| Dietary cholesterol | -0.01 | 0.0075000 | (Liu et al., 2002) (their Table 1) |
| Energy intake | 0.001 | 0.0007500 | (Liu et al., 2002; Platz et al., 1997) |
| Wave | 0 | 1 | (Casagrande et al., 2007) |
| Wave^2^ | 0 | 1 |  |

The γ shown was used for all three PFAS studied.

References

Bolton-Smith, C., Woodward, M., Smith, W.C., Tunstall-Pedoe, H., 1991. Dietary and non-dietary predictors of serum total and HDL-cholesterol in men and women: results from the Scottish Heart Health Study. Int. J. Epidemiol. 20, 95–104. https://doi.org/10.1093/ije/20.1.95

Carroll, M.D., 2020. Total and High-density Lipoprotein Cholesterol in Adults: United States, 2015–2018 8.

Casagrande, S.S., Wang, Y., Anderson, C., Gary, T.L., 2007. Have Americans increased their fruit and vegetable intake? The trends between 1988 and 2002. Am. J. Prev. Med. 32, 257–263. https://doi.org/10.1016/j.amepre.2006.12.002

Dzierlenga, M.W., Keast, D.R., Longnecker, M.P., 2021. The concentration of several perfluoroalkyl acids in serum appears to be reduced by dietary fiber. Environ. Int. 146, 106292. https://doi.org/10.1016/j.envint.2020.106292

Frank, A.T.H., Zhao, B., Jose, P.O., Azar, K.M.J., Fortmann, S.P., Palaniappan, L.P., 2014. Racial/ethnic differences in dyslipidemia patterns. Circulation 129, 570–579. https://doi.org/10.1161/CIRCULATIONAHA.113.005757

Fuchs, C.S., Giovannucci, E.L., Colditz, G.A., Hunter, D.J., Stampfer, M.J., Rosner, B., Speizer, F.E., Willett, W.C., 1999. Dietary fiber and the risk of colorectal cancer and adenoma in women. N. Engl. J. Med. 340, 169–176. https://doi.org/10.1056/NEJM199901213400301

Keto, J., Ventola, H., Jokelainen, J., Linden, K., Keinänen-Kiukaanniemi, S., Timonen, M., Ylisaukko-Oja, T., Auvinen, J., 2016. Cardiovascular disease risk factors in relation to smoking behaviour and history: a population-based cohort study. Open Heart 3, e000358. https://doi.org/10.1136/openhrt-2015-000358

Lanza, E., Jones, D.Y., Block, G., Kessler, L., 1987. Dietary fiber intake in the US population. Am. J. Clin. Nutr. 46, 790–797. https://doi.org/10.1093/ajcn/46.5.790

Liu, S., Buring, J.E., Sesso, H.D., Rimm, E.B., Willett, W.C., Manson, J.E., 2002. A prospective study of dietary fiber intake and risk of cardiovascular disease among women. J. Am. Coll. Cardiol. 39, 49–56. https://doi.org/10.1016/s0735-1097(01)01695-3

Luepker, R.V., Rosamond, W.D., Murphy, R., Sprafka, J.M., Folsom, A.R., McGovern, P.G., Blackburn, H., 1993. Socioeconomic status and coronary heart disease risk factor trends. The Minnesota Heart Survey. Circulation 88, 2172–2179. https://doi.org/10.1161/01.cir.88.5.2172

Platz, E.A., Giovannucci, E., Rimm, E.B., Rockett, H.R., Stampfer, M.J., Colditz, G.A., Willett, W.C., 1997. Dietary fiber and distal colorectal adenoma in men. Cancer Epidemiol. Biomark. Prev. Publ. Am. Assoc. Cancer Res. Cosponsored Am. Soc. Prev. Oncol. 6, 661–670.

Schneider, K.A., Heyden, S., Ford, C., 1987. Failure to reduce cholesterol as explanation for the limited efficacy of antihypertensive treatment in the reduction of coronary heart disease. Evidence from the Hypertension Detection and Follow-up program (1973-1979). Nephron 47 Suppl 1, 104–107. https://doi.org/10.1159/000184564

Seidell, J.C., Cigolini, M., Charzewska, J., Ellsinger, B.M., Björntorp, P., Hautvast, J.G., Szostak, W., 1991. Fat distribution and gender differences in serum lipids in men and women from four European communities. Atherosclerosis 87, 203–210. https://doi.org/10.1016/0021-9150(91)90022-u

Sonnenberg, L.M., Posner, B.M., Belanger, A.J., Cupples, L.A., D’Agostino, R.B., 1992. Dietary predictors of serum cholesterol in men: the Framingham cohort population. J. Clin. Epidemiol. 45, 413–418. https://doi.org/10.1016/0895-4356(92)90042-l

Subar, A.F., Harlan, L.C., Mattson, M.E., 1990. Food and nutrient intake differences between smokers and non-smokers in the US. Am. J. Public Health 80, 1323–1329. https://doi.org/10.2105/ajph.80.11.1323

# Supplemental Figures


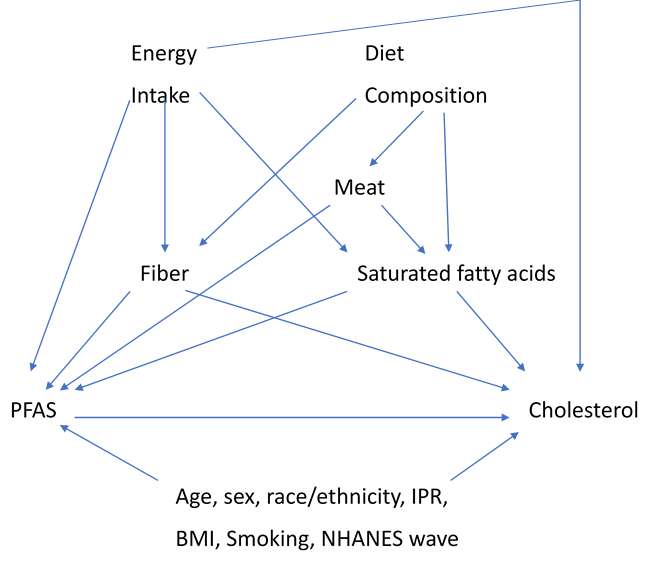


Figure S1. Directed acyclic graph depicting the conceptual model underlying the statistical model of serum cholesterol concentration. While the absolute amount of fiber and saturated fatty acids are of importance in determining serum cholesterol or serum PFAS concentration, from a practical standpoint it is only diet composition that can be manipulated. Thus, fiber and saturated fatty acids are energy-adjusted before inclusion in the model. In the figure meat could be replaced by other food items such as dairy that would be expected to have a positive relation to PFAS and serum cholesterol. Similarly, saturated fatty acids could be replaced by dietary cholesterol. Note that the effect on serum cholesterol of meat and similar items is mediated entirely through saturated fatty acids or dietary cholesterol. (Ingestion of plant sterols could interfere with absorption of dietary cholesterol, but dietary intake data on this are not available, and this component of diet likely has a negligible effect on serum cholesterol in a general population.) Because all the effect of meat or dairy on serum cholesterol is mediated by saturated fatty acids or dietary cholesterol, it is not necessary to adjust for meat or dairy in the statistical model. Although we included energy intake in the statistical model, inclusion was not necessary because nutrient intake (fiber, saturated fatty acids, dietary cholesterol) were all energy adjusted. Inclusion of energy intake, however, would not harm the aspect of the model of specific interest of us, and makes it a better general purpose model of serum cholesterol because it captures the effect of physical activity on total serum cholesterol, which includes high density lipoprotein cholesterol (arrow not shown). The potential effect of measurement error in intake of saturated fatty acids, etc. on our assessment of confounding by fiber of the cholesterol-PFAS association is discussed in the main text.

# Supplemental Tables

Table S1. Characteristics of the 7,242 NHANES subjects included in the analysis

| Characteristic | Median or number (percent) | Quartiles |
| --- | --- | --- |
| Serum cholesterol (mg/dL) | 197 | 171-224 |
| Energy intake (kcal/d) | 1915 | 1490-2487 |
| Saturated fat intake (g/d) | 22.8 | 15.7-31.2 |
| Dietary cholesterol intake (mg/d) | 244 | 157-375 |
| Age (y) | 44 | 32-59 |
| Sex |  |  |
| Females | 3973 (54.9%) |  |
| Males | 3269 (45.1%) |  |
| Race/Ethnicity |  |  |
| Mexican American | 1230 (17.0%) |  |
| Other Hispanic | 665 (9.2%) |  |
| Non-Hispanic White | 3234 (44.7%) |  |
| Non-Hispanic Black | 1522 (21.0%) |  |
| Other | 591 (8.1%) |  |
| Income to poverty ratio | 2.26 | 1.20-3.92 |
| Body mass index (kg/m^2^) | 32.7 | 29.0-37.3 |
| Smoking |  |  |
| Never smoker  Former smoker  Current smoker, < 1 pack/d  Current smoker, ≥ 1 pack/d | 4149 (57.3%)  1589 (21.9%)  1359 (18.8%)  145 (2.0%) |  |
| Energy-adjusted total fiber intake (g/d) | 14.9 | 10.5-20.7 |
| Energy-adjusted soluble fiber intake (g/d) | 4.39 | 3.08-6.09 |
| PFOA (ng/ml) | 2.7 | 1.7-4.4 |
| PFOS (ng/ml) | 9.9 | 5.1-18 |
| PFNA (ng/ml) | 0.90 | 0.60-1.43 |

* In the models smoking was coded as an ordinal variable; 0, never smoker; 1, former smoker; 2, current smoker (< 1 pack/d); 3, current smoker (≥ 1 pack/d).

Table S2: Coefficients^a^ (error estimates^b^) for fiber(ln[g/d])^c^ and PFOA (ng/ml) in multivariable models^d^ of ln(serum cholesterol), according to fiber variable included in model and type of model; n = 7,242.

| **Fiber variable^c^ included in model** | **ln-transformed energy intake-adjusted fiber (ln[g/d])** | | **PFAS (ng/ml)** | |
| --- | --- | --- | --- | --- |
|  | OLS | Bayesian | OLS | Bayesian |
| PFOS |  |  |  |  |
| None | N/A | N/A | 8.40 (1.6) | 8.38 (1.6) |
| Total fiber | -1.78 (1.58) | -3.55 (3.22) | 8.28 (1.6) | 8.08 (1.6) |
| Soluble fiber | -2.77 (1.06) | -3.46 (1.50) | 8.10 (1.6) | 8.12 (1.6) |
| PFNA |  |  |  |  |
| None | N/A | N/A | 8.35 (2.0) | 6.98 (1.6) |
| Total fiber | -1.79 (1.58) | -2.36 (1.57) | 8.18 (2.0) | 6.87 (1.7) |
| Soluble fiber | -2.76 (1.06) | -3.59 (1.52) | 7.88 (2.0) | 6.60 (1.6) |

^a^ To simplify the presentation coefficients and error estimates were multiplied by 10^-4^ for PFOS, 10^-3^ for PFNA, and 10^-2^ for fiber

^b^ Ordinary least squares (OLS) error estimates are standard errors and Bayesian error estimates are standard deviations

^c^ Modeled as ln-transformed energy intake-adjusted fiber

^d^ All models were adjusted for the secondary covariates energy intake, saturated fat intake, dietary cholesterol intake, age, sex, race/ethnicity (5 categories), income to poverty ratio, smoking (4 categories, treated as an ordinal variable), and a linear and a quadratic wave variable

Table S3A. Comparison of model coefficients from ordinary least squares (n = 7,242) and Stan (n = 7,242) model with no fiber adjustment for PFOS.

|  | R Linear Model | | | Stan | | Ratios | |
| --- | --- | --- | --- | --- | --- | --- | --- |
|  | β | SE(β) | T | β | SD(β) | β ratio  (LM/Stan) | SE/SD ratio  (LM/Stan) |
| (Intercept) | 5.27E+00 | 7.22E-03 | 729.756 | 5.27E+00 | 7.10E-03 | 1.00 | 1.02 |
| PFOS | 8.40E-04 | 1.62E-04 | 5.188 | 8.38E-04 | 1.62E-04 | 1.00 | 1.00 |
| Energy Intake | 2.40E-07 | 3.10E-06 | 0.077 | 2.00E-07 | 3.10E-06 | 1.20 | 1.00 |
| Saturated Fat Intake | 1.03E-04 | 3.17E-04 | 0.326 | 9.84E-05 | 3.12E-04 | 1.05 | 1.02 |
| Dietary Cholesterol | 3.87E-06 | 1.48E-05 | 0.261 | 3.90E-06 | 1.46E-05 | 0.99 | 1.02 |
| Age | 2.79E-03 | 1.38E-04 | 20.186 | 2.79E-03 | 1.40E-04 | 1.00 | 0.99 |
| Sex | 2.93E-02 | 5.00E-03 | 5.853 | 2.91E-02 | 4.87E-03 | 1.01 | 1.03 |
| Mexican American | 1.83E-02 | 6.65E-03 | 2.749 | 1.80E-02 | 6.59E-03 | 1.02 | 1.01 |
| Other Hispanic | 2.94E-02 | 8.27E-03 | 3.553 | 2.91E-02 | 8.26E-03 | 1.01 | 1.00 |
| Non-Hispanic Black | -3.21E-02 | 6.07E-03 | -5.294 | -3.23E-02 | 6.02E-03 | 0.99 | 1.01 |
| Other Race | 3.10E-03 | 8.78E-03 | 0.353 | 2.99E-03 | 8.91E-03 | 1.04 | 0.99 |
| Income-to-poverty ratio | 6.57E-03 | 1.48E-03 | 4.446 | 6.55E-03 | 1.48E-03 | 1.00 | 1.00 |
| BMI | 1.71E-03 | 3.26E-04 | 5.26 | 1.72E-03 | 3.21E-04 | 1.00 | 1.01 |
| Smoker | 9.83E-03 | 2.69E-03 | 3.656 | 9.74E-03 | 2.68E-03 | 1.01 | 1.00 |
| Wave | -1.95E-03 | 4.10E-03 | -0.476 | -2.01E-03 | 4.08E-03 | 0.97 | 1.01 |
| Wave^2^ | -5.71E-04 | 6.45E-04 | -0.885 | -5.67E-04 | 6.41E-04 | 1.01 | 1.01 |
| Model error | MSE | 0.1873 |  | σ(lnchol) | 0.1873 |  |  |

Table S3B. Comparison of model coefficients from ordinary least squares (n = 7,242) and Stan (n = 7,242) model with overall fiber for PFOS.

|  | R Linear Model | | | Stan | | Ratios | |
| --- | --- | --- | --- | --- | --- | --- | --- |
|  | β | SE(β) | T | β | SD(β) | β ratio  (LM/Stan) | SE/SD ratio  (LM/Stan) |
| (Intercept) | 5.27E+00 | 7.22E-03 | 729.533 | 5.27E+00 | 7.28E-03 | 1.00 | 0.99 |
| PFOS | 8.28E-04 | 1.62E-04 | 5.106 | 8.08E-04 | 1.64E-04 | 1.03 | 0.99 |
| Total Fiber | -1.78E-02 | 1.58E-02 | -1.127 | -3.55E-02 | 3.22E-02 | 0.50 | 0.49 |
| Energy Intake | 2.80E-07 | 3.10E-06 | 0.09 | 4.00E-07 | 3.10E-06 | 0.70 | 1.00 |
| Saturated Fat Intake | 4.15E-05 | 3.22E-04 | 0.129 | -1.68E-05 | 3.31E-04 | -2.47 | 0.97 |
| Dietary Cholesterol | 1.50E-06 | 1.50E-05 | 0.1 | -1.00E-06 | 1.53E-05 | -1.50 | 0.98 |
| Age | 2.82E-03 | 1.41E-04 | 19.961 | 2.85E-03 | 1.50E-04 | 0.99 | 0.94 |
| Sex | 2.95E-02 | 5.00E-03 | 5.897 | 2.95E-02 | 4.94E-03 | 1.00 | 1.01 |
| Mexican American | 1.96E-02 | 6.76E-03 | 2.904 | 2.08E-02 | 7.01E-03 | 0.94 | 0.96 |
| Other Hispanic | 2.95E-02 | 8.27E-03 | 3.568 | 2.95E-02 | 8.20E-03 | 1.00 | 1.01 |
| Non-Hispanic Black | -3.29E-02 | 6.11E-03 | -5.387 | -3.37E-02 | 6.12E-03 | 0.98 | 1.00 |
| Other Race | 3.51E-03 | 8.79E-03 | 0.399 | 3.92E-03 | 8.75E-03 | 0.90 | 1.00 |
| Income-to-poverty ratio | 6.73E-03 | 1.49E-03 | 4.531 | 6.91E-03 | 1.52E-03 | 0.97 | 0.98 |
| BMI | 1.69E-03 | 3.26E-04 | 5.192 | 1.68E-03 | 3.27E-04 | 1.01 | 1.00 |
| Smoker | 9.23E-03 | 2.74E-03 | 3.371 | 8.56E-03 | 2.93E-03 | 1.08 | 0.94 |
| Wave | -1.76E-03 | 4.10E-03 | -0.428 | -1.83E-03 | 4.08E-03 | 0.96 | 1.01 |
| Wave^2^ | -5.89E-04 | 6.45E-04 | -0.913 | -5.73E-04 | 6.39E-04 | 1.03 | 1.01 |
| Model error | MSE | 0.1872 |  | σ(lnchol) | 0.1872 |  |  |

Table S3C. Comparison of model coefficients from ordinary least squares (n = 7,242) and Stan (n = 7,242) model with soluble fiber for PFOS.

|  | R Linear Model | | | Stan | | Ratios | |
| --- | --- | --- | --- | --- | --- | --- | --- |
|  | β | SE(β) | T | β | SD(β) | β ratio  (LM/Stan) | SE/SD ratio  (LM/Stan) |
| (Intercept) | 5.27E+00 | 7.22E-03 | 729.977 | 5.27E+00 | 7.03E-03 | 1.00 | 1.03 |
| PFOS | 8.10E-04 | 1.62E-04 | 4.992 | 8.12E-04 | 1.61E-04 | 1.00 | 1.01 |
| Soluble Fiber | -2.77E-02 | 1.06E-02 | -2.613 | -3.46E-02 | 1.50E-02 | 0.80 | 0.71 |
| Energy Intake | -3.90E-08 | 3.11E-06 | -0.013 | -1.00E-06 | 3.00E-06 | 0.04 | 1.04 |
| Saturated Fat Intake | 4.16E-05 | 3.18E-04 | 0.131 | 1.47E-04 | 2.90E-04 | 0.28 | 1.10 |
| Dietary Cholesterol | -1.46E-06 | 1.50E-05 | -0.097 | 9.00E-07 | 1.41E-05 | -1.62 | 1.06 |
| Age | 2.84E-03 | 1.40E-04 | 20.356 | 2.84E-03 | 1.39E-04 | 1.00 | 1.00 |
| Sex | 2.95E-02 | 5.00E-03 | 5.906 | 2.96E-02 | 5.00E-03 | 1.00 | 1.00 |
| Mexican American | 2.15E-02 | 6.77E-03 | 3.183 | 2.25E-02 | 6.75E-03 | 0.96 | 1.00 |
| Other Hispanic | 2.93E-02 | 8.26E-03 | 3.55 | 2.94E-02 | 8.07E-03 | 1.00 | 1.02 |
| Non-Hispanic Black | -3.40E-02 | 6.11E-03 | -5.56 | -3.43E-02 | 6.15E-03 | 0.99 | 0.99 |
| Other Race | 2.84E-03 | 8.78E-03 | 0.324 | 3.12E-03 | 8.94E-03 | 0.91 | 0.98 |
| Income-to-poverty ratio | 6.73E-03 | 1.48E-03 | 4.551 | 6.85E-03 | 1.46E-03 | 0.98 | 1.01 |
| BMI | 1.68E-03 | 3.26E-04 | 5.17 | 1.68E-03 | 3.16E-04 | 1.00 | 1.03 |
| Smoker | 8.68E-03 | 2.72E-03 | 3.187 | 9.94E-03 | 2.52E-03 | 0.87 | 1.08 |
| Wave | -1.59E-03 | 4.10E-03 | -0.387 | -1.48E-03 | 4.09E-03 | 1.07 | 1.00 |
| Wave^2^ | -6.03E-04 | 6.45E-04 | -0.935 | -6.13E-04 | 6.44E-04 | 0.98 | 1.00 |
| Model error | MSE | 0.1872 |  | σ(lnchol) | 0.1872 |  |  |

Table S4A. Comparison of model coefficients from ordinary least squares (n = 7,242) and Stan (n = 7,242) model with no fiber adjustment for PFOA.

|  | R lm | | | Stan | | Ratios | |
| --- | --- | --- | --- | --- | --- | --- | --- |
|  | β | SE(β) | T | β | SD(β) | β ratio  (R/Stan) | SE/SD ratio  (R/Stan) |
| (Intercept) | 5.28E+00 | 6.98E-03 | 755.623 | 5.28E+00 | 6.81E-03 | 1.00 | 1.02 |
| PFOA | 2.59E-03 | 7.45E-04 | 3.479 | 2.66E-03 | 7.11E-04 | 0.97 | 1.05 |
| Energy Intake | -3.77E-07 | 3.11E-06 | -0.121 | -1.30E-06 | 3.10E-06 | 0.29 | 1.00 |
| Saturated Fat Intake | 6.23E-05 | 3.17E-04 | 0.196 | 1.81E-04 | 2.91E-04 | 0.34 | 1.09 |
| Dietary Cholesterol | 6.11E-06 | 1.48E-05 | 0.412 | 9.00E-06 | 1.41E-05 | 0.68 | 1.05 |
| Age | 2.89E-03 | 1.36E-04 | 21.28 | 2.88E-03 | 1.39E-04 | 1.00 | 0.98 |
| Sex | 2.67E-02 | 4.96E-03 | 5.388 | 2.66E-02 | 4.92E-03 | 1.00 | 1.01 |
| Mexican American | 1.78E-02 | 6.67E-03 | 2.673 | 1.81E-02 | 6.75E-03 | 0.98 | 0.99 |
| Other Hispanic | 2.85E-02 | 8.27E-03 | 3.44 | 2.89E-02 | 8.28E-03 | 0.98 | 1.00 |
| Non-Hispanic Black | -2.82E-02 | 6.04E-03 | -4.672 | -2.80E-02 | 6.02E-03 | 1.01 | 1.00 |
| Other Race | 5.58E-03 | 8.79E-03 | 0.635 | 5.86E-03 | 8.80E-03 | 0.95 | 1.00 |
| Income-to-poverty ratio | 6.43E-03 | 1.48E-03 | 4.335 | 6.53E-03 | 1.52E-03 | 0.98 | 0.98 |
| BMI | 1.68E-03 | 3.26E-04 | 5.166 | 1.69E-03 | 3.27E-04 | 1.00 | 1.00 |
| Smoker | 9.20E-03 | 2.69E-03 | 3.42 | 1.06E-02 | 2.43E-03 | 0.87 | 1.11 |
| Wave | -5.74E-03 | 4.03E-03 | -1.424 | -5.93E-03 | 4.03E-03 | 0.97 | 1.00 |
| Wave^2^ | -1.83E-04 | 6.45E-04 | -0.283 | -1.51E-04 | 6.47E-04 | 1.21 | 1.00 |
| Model error | MSE | 0.1875 |  | σ(lnchol) | 0.1875 |  |  |

Table S4B. Comparison of model coefficients from ordinary least squares (n = 7,242) and Stan (n = 7,242) model with overall fiber for PFOA.

|  | R lm | | | Stan | | Ratios | |
| --- | --- | --- | --- | --- | --- | --- | --- |
|  | β | SE(β) | T | β | SD(β) | β ratio  (R/Stan) | SE/SD ratio  (R/Stan) |
| (Intercept) | 5.28E+00 | 6.99E-03 | 755.206 | 5.27E+00 | 6.81E-03 | 1.00 | 1.03 |
| PFOA | 2.53E-03 | 7.47E-04 | 3.387 | 2.59E-03 | 7.15E-04 | 0.98 | 1.04 |
| Total Fiber | -1.92E-02 | 1.58E-02 | -1.216 | -2.35E-02 | 1.58E-02 | 0.82 | 1.00 |
| Energy Intake | -3.24E-07 | 3.11E-06 | -0.104 | -1.20E-06 | 3.00E-06 | 0.27 | 1.04 |
| Saturated Fat Intake | -3.90E-06 | 3.22E-04 | -0.012 | 1.10E-04 | 2.94E-04 | -0.04 | 1.09 |
| Dietary Cholesterol | 3.54E-06 | 1.50E-05 | 0.236 | 5.70E-06 | 1.41E-05 | 0.62 | 1.06 |
| Age | 2.92E-03 | 1.39E-04 | 21.058 | 2.92E-03 | 1.35E-04 | 1.00 | 1.03 |
| Sex | 2.70E-02 | 4.97E-03 | 5.44 | 2.71E-02 | 4.94E-03 | 1.00 | 1.00 |
| Mexican American | 1.93E-02 | 6.77E-03 | 2.843 | 1.99E-02 | 6.67E-03 | 0.97 | 1.02 |
| Other Hispanic | 2.86E-02 | 8.27E-03 | 3.457 | 2.89E-02 | 8.14E-03 | 0.99 | 1.02 |
| Non-Hispanic Black | -2.91E-02 | 6.08E-03 | -4.785 | -2.91E-02 | 6.06E-03 | 1.00 | 1.00 |
| Other Race | 5.98E-03 | 8.79E-03 | 0.68 | 6.45E-03 | 8.80E-03 | 0.93 | 1.00 |
| Income-to-poverty ratio | 6.61E-03 | 1.49E-03 | 4.432 | 6.76E-03 | 1.50E-03 | 0.98 | 0.99 |
| BMI | 1.66E-03 | 3.26E-04 | 5.094 | 1.67E-03 | 3.19E-04 | 0.99 | 1.02 |
| Smoker | 8.57E-03 | 2.74E-03 | 3.129 | 9.96E-03 | 2.47E-03 | 0.86 | 1.11 |
| Wave | -5.47E-03 | 4.03E-03 | -1.356 | -5.44E-03 | 4.06E-03 | 1.01 | 0.99 |
| Wave^2^ | -2.09E-04 | 6.45E-04 | -0.324 | -2.08E-04 | 6.50E-04 | 1.01 | 0.99 |
| Model error | MSE | 0.1875 |  | σ(lnchol) | 0.1875 |  |  |

Table S4C. Comparison of model coefficients from OLS (n = 7242) and Stan (n = 7242) model with soluble fiber for PFOA.

|  | R lm | | | Stan | | Ratios | |
| --- | --- | --- | --- | --- | --- | --- | --- |
|  | β | SE(β) | T | β | SD(β) | β ratio  (R/Stan) | SE/SD ratio  (R/Stan) |
| (Intercept) | 5.28E+00 | 6.98E-03 | 755.733 | 5.27E+00 | 6.87E-03 | 1.00 | 1.02 |
| PFOA | 2.45E-03 | 7.47E-04 | 3.278 | 2.49E-03 | 7.15E-04 | 0.98 | 1.04 |
| Soluble Fiber | -2.90E-02 | 1.06E-02 | -2.731 | -3.53E-02 | 1.49E-02 | 0.82 | 0.71 |
| Energy Intake | -6.45E-07 | 3.11E-06 | -0.208 | -1.60E-06 | 3.00E-06 | 0.40 | 1.04 |
| Saturated Fat Intake | -8.41E-07 | 3.18E-04 | -0.003 | 1.08E-04 | 2.88E-04 | -0.01 | 1.11 |
| Dietary Cholesterol | 4.87E-07 | 1.50E-05 | 0.033 | 2.70E-06 | 1.45E-05 | 0.18 | 1.03 |
| Age | 2.95E-03 | 1.37E-04 | 21.459 | 2.94E-03 | 1.37E-04 | 1.00 | 1.00 |
| Sex | 2.71E-02 | 4.96E-03 | 5.454 | 2.72E-02 | 4.91E-03 | 0.99 | 1.01 |
| Mexican American | 2.12E-02 | 6.78E-03 | 3.126 | 2.23E-02 | 6.77E-03 | 0.95 | 1.00 |
| Other Hispanic | 2.84E-02 | 8.27E-03 | 3.439 | 2.87E-02 | 8.16E-03 | 0.99 | 1.01 |
| Non-Hispanic Black | -3.03E-02 | 6.08E-03 | -4.977 | -3.05E-02 | 5.94E-03 | 0.99 | 1.02 |
| Other Race | 5.20E-03 | 8.78E-03 | 0.592 | 5.70E-03 | 8.62E-03 | 0.91 | 1.02 |
| Income-to-poverty ratio | 6.61E-03 | 1.49E-03 | 4.454 | 6.76E-03 | 1.49E-03 | 0.98 | 1.00 |
| BMI | 1.65E-03 | 3.26E-04 | 5.074 | 1.65E-03 | 3.28E-04 | 1.00 | 0.99 |
| Smoker | 8.03E-03 | 2.72E-03 | 2.947 | 9.44E-03 | 2.54E-03 | 0.85 | 1.07 |
| Wave | -5.22E-03 | 4.03E-03 | -1.294 | -5.20E-03 | 4.09E-03 | 1.00 | 0.99 |
| Wave^2^ | -2.34E-04 | 6.45E-04 | -0.362 | -2.30E-04 | 6.47E-04 | 1.02 | 1.00 |
| Model error | MSE | 0.1874 |  | σ(lnchol) | 0.1874 |  |  |

Table S5A. Comparison of model coefficients from OLS (n = 7242) and Stan (n = 7242) model with no adjustment for fiber for PFNA.

|  | R lm | | | Stan | | Ratios | |
| --- | --- | --- | --- | --- | --- | --- | --- |
|  | β | SE(β) | T | β | SD(β) | β ratio  (R/Stan) | SE/SD ratio  (R/Stan) |
| (Intercept) | 5.28E+00 | 6.86E-03 | 769.721 | 5.28E+00 | 6.84E-03 | 1.00 | 1.00 |
| PFNA | 8.35E-03 | 1.98E-03 | 4.227 | 6.98E-03 | 1.63E-03 | 1.20 | 1.21 |
| Energy Intake | -1.24E-07 | 3.10E-06 | -0.04 | -1.00E-06 | 3.10E-06 | 0.12 | 1.00 |
| Saturated Fat Intake | 1.08E-04 | 3.17E-04 | 0.342 | 2.07E-04 | 3.02E-04 | 0.52 | 1.05 |
| Dietary Cholesterol | 3.68E-06 | 1.48E-05 | 0.248 | 7.70E-06 | 1.43E-05 | 0.48 | 1.04 |
| Age | 2.88E-03 | 1.36E-04 | 21.238 | 2.88E-03 | 1.34E-04 | 1.00 | 1.01 |
| Sex | 2.62E-02 | 4.93E-03 | 5.317 | 2.60E-02 | 4.94E-03 | 1.01 | 1.00 |
| Mexican American | 1.72E-02 | 6.65E-03 | 2.589 | 1.72E-02 | 6.49E-03 | 1.00 | 1.02 |
| Other Hispanic | 2.69E-02 | 8.26E-03 | 3.258 | 2.75E-02 | 8.18E-03 | 0.98 | 1.01 |
| Non-Hispanic Black | -3.06E-02 | 6.05E-03 | -5.053 | -3.00E-02 | 5.96E-03 | 1.02 | 1.02 |
| Other Race | 3.01E-03 | 8.79E-03 | 0.342 | 3.92E-03 | 8.62E-03 | 0.77 | 1.02 |
| Income-to-poverty ratio | 6.49E-03 | 1.48E-03 | 4.382 | 6.69E-03 | 1.48E-03 | 0.97 | 1.00 |
| BMI | 1.67E-03 | 3.26E-04 | 5.133 | 1.69E-03 | 3.27E-04 | 0.99 | 1.00 |
| Smoker | 9.38E-03 | 2.69E-03 | 3.488 | 1.09E-02 | 2.41E-03 | 0.86 | 1.11 |
| Wave | -7.63E-03 | 4.04E-03 | -1.888 | -7.51E-03 | 4.05E-03 | 1.02 | 1.00 |
| Wave^2^ | 7.26E-05 | 6.50E-04 | 0.112 | 2.37E-05 | 6.52E-04 | 3.06 | 1.00 |
| Model error | MSE | 0.1874 |  | σ(lnchol) | 0.1874 |  |  |

Table S5B. Comparison of model coefficients from OLS (n = 7242) and Stan (n = 7242) model with overall fiber for PFNA.

|  | R lm | | | Stan | | Ratios | |
| --- | --- | --- | --- | --- | --- | --- | --- |
|  | β | SE(β) | T | β | SD(β) | β ratio  (R/Stan) | SE/SD ratio  (R/Stan) |
| (Intercept) | 5.28E+00 | 6.87E-03 | 768.948 | 5.28E+00 | 6.76E-03 | 1.00 | 1.02 |
| PFNA | 8.18E-03 | 1.98E-03 | 4.128 | 6.87E-03 | 1.69E-03 | 1.19 | 1.17 |
| Total Fiber | -1.79E-02 | 1.58E-02 | -1.133 | -2.36E-02 | 1.57E-02 | 0.76 | 1.00 |
| Energy Intake | -7.95E-08 | 3.11E-06 | -0.026 | -1.00E-06 | 3.10E-06 | 0.08 | 1.00 |
| Saturated Fat Intake | 4.57E-05 | 3.22E-04 | 0.142 | 1.39E-04 | 2.99E-04 | 0.33 | 1.08 |
| Dietary Cholesterol | 1.33E-06 | 1.50E-05 | 0.088 | 4.80E-06 | 1.43E-05 | 0.28 | 1.05 |
| Age | 2.91E-03 | 1.39E-04 | 20.997 | 2.92E-03 | 1.40E-04 | 1.00 | 0.99 |
| Sex | 2.65E-02 | 4.94E-03 | 5.367 | 2.63E-02 | 4.95E-03 | 1.01 | 1.00 |
| Mexican American | 1.86E-02 | 6.75E-03 | 2.748 | 1.90E-02 | 6.79E-03 | 0.98 | 0.99 |
| Other Hispanic | 2.71E-02 | 8.26E-03 | 3.277 | 2.74E-02 | 8.09E-03 | 0.99 | 1.02 |
| Non-Hispanic Black | -3.14E-02 | 6.09E-03 | -5.148 | -3.11E-02 | 5.86E-03 | 1.01 | 1.04 |
| Other Race | 3.44E-03 | 8.80E-03 | 0.391 | 4.13E-03 | 8.93E-03 | 0.83 | 0.98 |
| Income-to-poverty ratio | 6.65E-03 | 1.49E-03 | 4.469 | 6.87E-03 | 1.48E-03 | 0.97 | 1.01 |
| BMI | 1.65E-03 | 3.26E-04 | 5.067 | 1.66E-03 | 3.31E-04 | 1.00 | 0.99 |
| Smoker | 8.79E-03 | 2.74E-03 | 3.208 | 1.01E-02 | 2.47E-03 | 0.87 | 1.11 |
| Wave | -7.35E-03 | 4.05E-03 | -1.814 | -7.03E-03 | 3.98E-03 | 1.04 | 1.02 |
| Wave^2^ | 4.28E-05 | 6.51E-04 | 0.066 | -3.07E-05 | 6.40E-04 | -1.39 | 1.02 |
| Model error | MSE | 0.1874 |  | σ(lnchol) |  |  |  |

Table S5C. Comparison of model coefficients from OLS (n = 7242) and Stan (n = 7242) model with soluble fiber for PFNA.

|  | R lm | | | Stan | | Ratios | |
| --- | --- | --- | --- | --- | --- | --- | --- |
|  | β | SE(β) | T | β | SD(β) | β ratio  (R/Stan) | SE/SD ratio  (R/Stan) |
| (Intercept) | 5.28E+00 | 6.86E-03 | 769.548 | 5.28E+00 | 6.68E-03 | 1.00 | 1.03 |
| PFNA | 7.88E-03 | 1.98E-03 | 3.974 | 6.60E-03 | 1.64E-03 | 1.19 | 1.21 |
| Soluble Fiber | -2.76E-02 | 1.06E-02 | -2.596 | -3.59E-02 | 1.52E-02 | 0.77 | 0.70 |
| Energy Intake | -3.93E-07 | 3.11E-06 | -0.127 | -1.30E-06 | 3.00E-06 | 0.30 | 1.04 |
| Saturated Fat Intake | 4.56E-05 | 3.18E-04 | 0.143 | 1.37E-04 | 2.97E-04 | 0.33 | 1.07 |
| Dietary Cholesterol | -1.53E-06 | 1.50E-05 | -0.102 | 1.40E-06 | 1.44E-05 | -1.09 | 1.04 |
| Age | 2.94E-03 | 1.37E-04 | 21.394 | 2.95E-03 | 1.42E-04 | 1.00 | 0.96 |
| Sex | 2.66E-02 | 4.93E-03 | 5.384 | 2.64E-02 | 4.87E-03 | 1.01 | 1.01 |
| Mexican American | 2.04E-02 | 6.76E-03 | 3.024 | 2.15E-02 | 6.72E-03 | 0.95 | 1.01 |
| Other Hispanic | 2.70E-02 | 8.26E-03 | 3.266 | 2.75E-02 | 8.24E-03 | 0.98 | 1.00 |
| Non-Hispanic Black | -3.24E-02 | 6.09E-03 | -5.321 | -3.23E-02 | 6.14E-03 | 1.00 | 0.99 |
| Other Race | 2.79E-03 | 8.79E-03 | 0.318 | 3.36E-03 | 8.67E-03 | 0.83 | 1.01 |
| Income-to-poverty ratio | 6.66E-03 | 1.48E-03 | 4.493 | 6.88E-03 | 1.49E-03 | 0.97 | 0.99 |
| BMI | 1.64E-03 | 3.26E-04 | 5.047 | 1.64E-03 | 3.27E-04 | 1.00 | 1.00 |
| Smoker | 8.25E-03 | 2.72E-03 | 3.03 | 9.54E-03 | 2.46E-03 | 0.86 | 1.11 |
| Wave | -7.03E-03 | 4.05E-03 | -1.738 | -6.61E-03 | 4.13E-03 | 1.06 | 0.98 |
| Wave^2^ | 9.27E-06 | 6.51E-04 | 0.014 | -7.01E-05 | 6.63E-04 | -0.13 | 0.98 |
| Model error | MSE | 0.1873 |  | σ(lnchol) | 0.1873 |  |  |
