# Supplementary material for: Is the cholesterol-perfluoroalkyl substance association confounded by dietary fiber intake?: a Bayesian analysis of NHANES data with adjustment for measurement error in fiber intake
Source: Environ Health. 2022 Nov 22;21:114. doi: 10.1186/s12940-022-00923-2 (PMC9682702; doi:10.1186/s12940-022-00923-2)
Supplement: Supplementary file 4 — Additional file 4. [file 12940_2022_923_MOESM4_ESM.zip › Supplemental_Code/pfas_fiber_bayes.nb.html]

R Notebook


Code 

- Show All Code
- Hide All Code
- Download Rmd

# R Notebook

# Stan Bayesian Model

This is the code to set up, load in, and run parameter fitting for
the Stan model. Please note that RTools must be installed for rstan to
run properly, and that it will throw a nonsensical error if there are
spaces in the file directory.

## Data Setup

Load in data


```
setwd(this.path::here())
indf <- read.csv("data_7-21-21.csv", header = T)
```


Now update data as needed


```
#Generate individual variables for ease of reference later
lnchol <- indf$lnchol 
pfos_c <- indf$adj_pfos
pfoa_c <- indf$adj_pfoa
pfna_c <- indf$adj_pfna
ei_c <- indf$adj_energy
lnu1_c <- indf$adj_lneafib1
lnu2_c <- indf$adj_lneafib2
lnus1_c <- indf$adj_lneafsol1
lnus2_c <- indf$adj_lneafsol2
dchol_c <- indf$adj_eadchol
sex <- indf$sex
satfat_c <- indf$adj_easatfat
age_c <- indf$adj_age
bmi_c <- indf$adj_bmi
eth1 <- indf$et1
eth2 <- indf$et2
eth3 <- indf$et4
eth4 <- indf$et5
#Make ethnicity variables into a  matrix
eth <- cbind(eth1, eth2, eth3, eth4)
#Center income to poverty variable on mean
indf$indfmpir_c <- indf$indfmpir - 2.545291
indfmpir_c <- indf$indfmpir_c
#Finish up with variable assignment
smoker <- indf$smoker
wave <- indf$wave
wave2 <- indf$wave2
N <- length(lnchol)
```


Now we need to set the settings for the run from the included .csv
file


```
settinginput <- read.csv("./Stan_settings.csv", header=TRUE)

iters <- settinginput$iters[!is.na(settinginput$iters)]
warmup_ratio <- settinginput$warmup_ratio[!is.na(settinginput$warmup_ratio)]
chains <- settinginput$chains[!is.na(settinginput$chains)]
thin <- settinginput$thin[!is.na(settinginput$thin)]

warmups <- iters * warmup_ratio
N_samples <- iters*chains*(1-warmup_ratio)/thin
```


Then we can set up some software options


```
library("rstan")
rstan_options(auto_write = TRUE)
options(mc.cores=parallel::detectCores())
```


Next we’ll read in the model code Note: The model code is where
you’ll need to change between different PFAS (PFOS, PFOA, PFNA), fiber
(none, total, soluble), or other variables as desired.


```
fiber_pars_code <- readChar("fiber_pars.txt",file.info("fiber_pars.txt")$size)
```


Then we’ll set an automated output file name for versioning


```
t <- format(Sys.time(), "%Y%m%d%H%M")
modname <- paste0("PFASfit_",t)
```

## Bayesian Model Fitting

Finally, we’re ready to run the model fit and save the output to a
file NOTE: THIS WILL TAKE AN EXTREMELY LONG TIME– LIKELY ON THE ORDER OF
DAYS UNLESS RUNNING ON SEVERAL CHAINS WITH A VERY STRONG PROCESSOR


```
#Run the model fit
starttime <- Sys.time()
fit_fiber_pars <- stan(model_name = modname, model_code=fiber_pars_code, data=c("N", "lnchol", "pfoa_c", "pfos_c", "pfna_c", "ei_c", "lnu1_c", "lnu2_c","lnus1_c", "lnus2_c", "dchol_c", "sex", "satfat_c", "age_c", "bmi_c", "eth", "indfmpir_c", "smoker", "wave", "wave2"), iter=iters, chains=chains, warmup=warmups, thin=thin, control=list(max_treedepth=15,adapt_delta=0.8), seed = 1234)
endtime <- Sys.time() - starttime

#Save the output
dir.create(paste0("Outputs/",t))
write.csv(In_data1, paste0("Outputs/",t,"/PFASdata_",t,".csv"))
write.table(fiber_pars_code, paste0("Outputs/",t,"/PFAScode_",t,".txt"),quote = F, row.names = F, col.names = F)
saveRDS(fit_fiber_pars,paste0("Outputs/",t,"/PFASfit_",t,".RDS"))
```

## Data Analysis

The output (fit\_fiber\_pars) can then either be pulled from the
variable above or imported back from the saved file for analysis.


```
#Read in from the file if needed
#fit_fiber_pars <- readRDS("FILENAME/FILEPATH.RDS")

#Check the fit values
print(fit_fiber_pars, pars = c("li"), include = FALSE, digit=7)

#Check run diagnostics
rstan::check_hmc_diagnostics(fit_fiber_pars)
#Generate a fit summary
fit_summary <- summary(fit_fiber_pars)
#Determine the Rhat spread and which variables have the largest and smallest Rhat values
summary(fit_summary$summary[,"Rhat"])
which.min(fit_summary$summary[,"Rhat"])
which.max(fit_summary$summary[,"Rhat"])
#Determine the effective n spread and which variables have the largest and smallest n_eff values
summary(fit_summary$summary[,"n_eff"])
which.min(fit_summary$summary[,"n_eff"])
which.max(fit_summary$summary[,"n_eff"])
#Determine run times (overall and for each chain)
rstan::get_elapsed_time(fit_fiber_pars)
sum(get_elapsed_time(fit_fiber_pars)[1,])/60/60
sum(get_elapsed_time(fit_fiber_pars)[2,])/60/60
sum(get_elapsed_time(fit_fiber_pars)[3,])/60/60
sum(get_elapsed_time(fit_fiber_pars)[4,])/60/60
```

LS0tDQp0aXRsZTogIlIgTm90ZWJvb2siDQpvdXRwdXQ6IGh0bWxfbm90ZWJvb2sNCi0tLQ0KDQojIFN0YW4gQmF5ZXNpYW4gTW9kZWwNCg0KVGhpcyBpcyB0aGUgY29kZSB0byBzZXQgdXAsIGxvYWQgaW4sIGFuZCBydW4gcGFyYW1ldGVyIGZpdHRpbmcgZm9yIHRoZSBTdGFuIG1vZGVsLiBQbGVhc2Ugbm90ZSB0aGF0IFJUb29scyBtdXN0IGJlIGluc3RhbGxlZCBmb3IgcnN0YW4gdG8gcnVuIHByb3Blcmx5LCBhbmQgdGhhdCBpdCB3aWxsIHRocm93IGEgbm9uc2Vuc2ljYWwgZXJyb3IgaWYgdGhlcmUgYXJlIHNwYWNlcyBpbiB0aGUgZmlsZSBkaXJlY3RvcnkuDQoNCiMjIERhdGEgU2V0dXANCg0KTG9hZCBpbiBkYXRhDQpgYGB7cn0NCnNldHdkKHRoaXMucGF0aDo6aGVyZSgpKQ0KaW5kZiA8LSByZWFkLmNzdigiZGF0YV83LTIxLTIxLmNzdiIsIGhlYWRlciA9IFQpDQpgYGANCg0KTm93IHVwZGF0ZSBkYXRhIGFzIG5lZWRlZA0KYGBge3J9DQojR2VuZXJhdGUgaW5kaXZpZHVhbCB2YXJpYWJsZXMgZm9yIGVhc2Ugb2YgcmVmZXJlbmNlIGxhdGVyDQpsbmNob2wgPC0gaW5kZiRsbmNob2wgDQpwZm9zX2MgPC0gaW5kZiRhZGpfcGZvcw0KcGZvYV9jIDwtIGluZGYkYWRqX3Bmb2ENCnBmbmFfYyA8LSBpbmRmJGFkal9wZm5hDQplaV9jIDwtIGluZGYkYWRqX2VuZXJneQ0KbG51MV9jIDwtIGluZGYkYWRqX2xuZWFmaWIxDQpsbnUyX2MgPC0gaW5kZiRhZGpfbG5lYWZpYjINCmxudXMxX2MgPC0gaW5kZiRhZGpfbG5lYWZzb2wxDQpsbnVzMl9jIDwtIGluZGYkYWRqX2xuZWFmc29sMg0KZGNob2xfYyA8LSBpbmRmJGFkal9lYWRjaG9sDQpzZXggPC0gaW5kZiRzZXgNCnNhdGZhdF9jIDwtIGluZGYkYWRqX2Vhc2F0ZmF0DQphZ2VfYyA8LSBpbmRmJGFkal9hZ2UNCmJtaV9jIDwtIGluZGYkYWRqX2JtaQ0KZXRoMSA8LSBpbmRmJGV0MQ0KZXRoMiA8LSBpbmRmJGV0Mg0KZXRoMyA8LSBpbmRmJGV0NA0KZXRoNCA8LSBpbmRmJGV0NQ0KI01ha2UgZXRobmljaXR5IHZhcmlhYmxlcyBpbnRvIGEgIG1hdHJpeA0KZXRoIDwtIGNiaW5kKGV0aDEsIGV0aDIsIGV0aDMsIGV0aDQpDQojQ2VudGVyIGluY29tZSB0byBwb3ZlcnR5IHZhcmlhYmxlIG9uIG1lYW4NCmluZGYkaW5kZm1waXJfYyA8LSBpbmRmJGluZGZtcGlyIC0gMi41NDUyOTENCmluZGZtcGlyX2MgPC0gaW5kZiRpbmRmbXBpcl9jDQojRmluaXNoIHVwIHdpdGggdmFyaWFibGUgYXNzaWdubWVudA0Kc21va2VyIDwtIGluZGYkc21va2VyDQp3YXZlIDwtIGluZGYkd2F2ZQ0Kd2F2ZTIgPC0gaW5kZiR3YXZlMg0KTiA8LSBsZW5ndGgobG5jaG9sKQ0KYGBgDQoNCk5vdyB3ZSBuZWVkIHRvIHNldCB0aGUgc2V0dGluZ3MgZm9yIHRoZSBydW4gZnJvbSB0aGUgaW5jbHVkZWQgLmNzdiBmaWxlDQpgYGB7cn0NCnNldHRpbmdpbnB1dCA8LSByZWFkLmNzdigiLi9TdGFuX3NldHRpbmdzLmNzdiIsIGhlYWRlcj1UUlVFKQ0KDQppdGVycyA8LSBzZXR0aW5naW5wdXQkaXRlcnNbIWlzLm5hKHNldHRpbmdpbnB1dCRpdGVycyldDQp3YXJtdXBfcmF0aW8gPC0gc2V0dGluZ2lucHV0JHdhcm11cF9yYXRpb1shaXMubmEoc2V0dGluZ2lucHV0JHdhcm11cF9yYXRpbyldDQpjaGFpbnMgPC0gc2V0dGluZ2lucHV0JGNoYWluc1shaXMubmEoc2V0dGluZ2lucHV0JGNoYWlucyldDQp0aGluIDwtIHNldHRpbmdpbnB1dCR0aGluWyFpcy5uYShzZXR0aW5naW5wdXQkdGhpbildDQoNCndhcm11cHMgPC0gaXRlcnMgKiB3YXJtdXBfcmF0aW8NCk5fc2FtcGxlcyA8LSBpdGVycypjaGFpbnMqKDEtd2FybXVwX3JhdGlvKS90aGluDQpgYGANCg0KVGhlbiB3ZSBjYW4gc2V0IHVwIHNvbWUgc29mdHdhcmUgb3B0aW9ucw0KYGBge3J9DQpsaWJyYXJ5KCJyc3RhbiIpDQpyc3Rhbl9vcHRpb25zKGF1dG9fd3JpdGUgPSBUUlVFKQ0Kb3B0aW9ucyhtYy5jb3Jlcz1wYXJhbGxlbDo6ZGV0ZWN0Q29yZXMoKSkNCmBgYA0KDQpOZXh0IHdlJ2xsIHJlYWQgaW4gdGhlIG1vZGVsIGNvZGUNCk5vdGU6IFRoZSBtb2RlbCBjb2RlIGlzIHdoZXJlIHlvdSdsbCBuZWVkIHRvIGNoYW5nZSBiZXR3ZWVuIGRpZmZlcmVudCBQRkFTIChQRk9TLCBQRk9BLCBQRk5BKSwgZmliZXIgKG5vbmUsIHRvdGFsLCBzb2x1YmxlKSwgb3Igb3RoZXIgdmFyaWFibGVzIGFzIGRlc2lyZWQuDQpgYGB7cn0NCmZpYmVyX3BhcnNfY29kZSA8LSByZWFkQ2hhcigiZmliZXJfcGFycy50eHQiLGZpbGUuaW5mbygiZmliZXJfcGFycy50eHQiKSRzaXplKQ0KYGBgDQoNClRoZW4gd2UnbGwgc2V0IGFuIGF1dG9tYXRlZCBvdXRwdXQgZmlsZSBuYW1lIGZvciB2ZXJzaW9uaW5nDQpgYGB7cn0NCnQgPC0gZm9ybWF0KFN5cy50aW1lKCksICIlWSVtJWQlSCVNIikNCm1vZG5hbWUgPC0gcGFzdGUwKCJQRkFTZml0XyIsdCkNCmBgYA0KDQojIyBCYXllc2lhbiBNb2RlbCBGaXR0aW5nDQoNCkZpbmFsbHksIHdlJ3JlIHJlYWR5IHRvIHJ1biB0aGUgbW9kZWwgZml0IGFuZCBzYXZlIHRoZSBvdXRwdXQgdG8gYSBmaWxlDQpOT1RFOiBUSElTIFdJTEwgVEFLRSBBTiBFWFRSRU1FTFkgTE9ORyBUSU1FLS0gTElLRUxZIE9OIFRIRSBPUkRFUiBPRiBEQVlTIFVOTEVTUyBSVU5OSU5HIE9OIFNFVkVSQUwgQ0hBSU5TIFdJVEggQSBWRVJZIFNUUk9ORyBQUk9DRVNTT1INCmBgYHtyfQ0KI1J1biB0aGUgbW9kZWwgZml0DQpzdGFydHRpbWUgPC0gU3lzLnRpbWUoKQ0KZml0X2ZpYmVyX3BhcnMgPC0gc3Rhbihtb2RlbF9uYW1lID0gbW9kbmFtZSwgbW9kZWxfY29kZT1maWJlcl9wYXJzX2NvZGUsIGRhdGE9YygiTiIsICJsbmNob2wiLCAicGZvYV9jIiwgInBmb3NfYyIsICJwZm5hX2MiLCAiZWlfYyIsICJsbnUxX2MiLCAibG51Ml9jIiwibG51czFfYyIsICJsbnVzMl9jIiwgImRjaG9sX2MiLCAic2V4IiwgInNhdGZhdF9jIiwgImFnZV9jIiwgImJtaV9jIiwgImV0aCIsICJpbmRmbXBpcl9jIiwgInNtb2tlciIsICJ3YXZlIiwgIndhdmUyIiksIGl0ZXI9aXRlcnMsIGNoYWlucz1jaGFpbnMsIHdhcm11cD13YXJtdXBzLCB0aGluPXRoaW4sIGNvbnRyb2w9bGlzdChtYXhfdHJlZWRlcHRoPTE1LGFkYXB0X2RlbHRhPTAuOCksIHNlZWQgPSAxMjM0KQ0KZW5kdGltZSA8LSBTeXMudGltZSgpIC0gc3RhcnR0aW1lDQoNCiNTYXZlIHRoZSBvdXRwdXQNCmRpci5jcmVhdGUocGFzdGUwKCJPdXRwdXRzLyIsdCkpDQp3cml0ZS5jc3YoSW5fZGF0YTEsIHBhc3RlMCgiT3V0cHV0cy8iLHQsIi9QRkFTZGF0YV8iLHQsIi5jc3YiKSkNCndyaXRlLnRhYmxlKGZpYmVyX3BhcnNfY29kZSwgcGFzdGUwKCJPdXRwdXRzLyIsdCwiL1BGQVNjb2RlXyIsdCwiLnR4dCIpLHF1b3RlID0gRiwgcm93Lm5hbWVzID0gRiwgY29sLm5hbWVzID0gRikNCnNhdmVSRFMoZml0X2ZpYmVyX3BhcnMscGFzdGUwKCJPdXRwdXRzLyIsdCwiL1BGQVNmaXRfIix0LCIuUkRTIikpDQpgYGANCg0KIyMgRGF0YSBBbmFseXNpcw0KDQpUaGUgb3V0cHV0IChmaXRfZmliZXJfcGFycykgY2FuIHRoZW4gZWl0aGVyIGJlIHB1bGxlZCBmcm9tIHRoZSB2YXJpYWJsZSBhYm92ZSBvciBpbXBvcnRlZCBiYWNrIGZyb20gdGhlIHNhdmVkIGZpbGUgZm9yIGFuYWx5c2lzLg0KYGBge3J9DQojUmVhZCBpbiBmcm9tIHRoZSBmaWxlIGlmIG5lZWRlZA0KI2ZpdF9maWJlcl9wYXJzIDwtIHJlYWRSRFMoIkZJTEVOQU1FL0ZJTEVQQVRILlJEUyIpDQoNCiNDaGVjayB0aGUgZml0IHZhbHVlcw0KcHJpbnQoZml0X2ZpYmVyX3BhcnMsIHBhcnMgPSBjKCJsaSIpLCBpbmNsdWRlID0gRkFMU0UsIGRpZ2l0PTcpDQoNCiNDaGVjayBydW4gZGlhZ25vc3RpY3MNCnJzdGFuOjpjaGVja19obWNfZGlhZ25vc3RpY3MoZml0X2ZpYmVyX3BhcnMpDQojR2VuZXJhdGUgYSBmaXQgc3VtbWFyeQ0KZml0X3N1bW1hcnkgPC0gc3VtbWFyeShmaXRfZmliZXJfcGFycykNCiNEZXRlcm1pbmUgdGhlIFJoYXQgc3ByZWFkIGFuZCB3aGljaCB2YXJpYWJsZXMgaGF2ZSB0aGUgbGFyZ2VzdCBhbmQgc21hbGxlc3QgUmhhdCB2YWx1ZXMNCnN1bW1hcnkoZml0X3N1bW1hcnkkc3VtbWFyeVssIlJoYXQiXSkNCndoaWNoLm1pbihmaXRfc3VtbWFyeSRzdW1tYXJ5WywiUmhhdCJdKQ0Kd2hpY2gubWF4KGZpdF9zdW1tYXJ5JHN1bW1hcnlbLCJSaGF0Il0pDQojRGV0ZXJtaW5lIHRoZSBlZmZlY3RpdmUgbiBzcHJlYWQgYW5kIHdoaWNoIHZhcmlhYmxlcyBoYXZlIHRoZSBsYXJnZXN0IGFuZCBzbWFsbGVzdCBuX2VmZiB2YWx1ZXMNCnN1bW1hcnkoZml0X3N1bW1hcnkkc3VtbWFyeVssIm5fZWZmIl0pDQp3aGljaC5taW4oZml0X3N1bW1hcnkkc3VtbWFyeVssIm5fZWZmIl0pDQp3aGljaC5tYXgoZml0X3N1bW1hcnkkc3VtbWFyeVssIm5fZWZmIl0pDQojRGV0ZXJtaW5lIHJ1biB0aW1lcyAob3ZlcmFsbCBhbmQgZm9yIGVhY2ggY2hhaW4pDQpyc3Rhbjo6Z2V0X2VsYXBzZWRfdGltZShmaXRfZmliZXJfcGFycykNCnN1bShnZXRfZWxhcHNlZF90aW1lKGZpdF9maWJlcl9wYXJzKVsxLF0pLzYwLzYwDQpzdW0oZ2V0X2VsYXBzZWRfdGltZShmaXRfZmliZXJfcGFycylbMixdKS82MC82MA0Kc3VtKGdldF9lbGFwc2VkX3RpbWUoZml0X2ZpYmVyX3BhcnMpWzMsXSkvNjAvNjANCnN1bShnZXRfZWxhcHNlZF90aW1lKGZpdF9maWJlcl9wYXJzKVs0LF0pLzYwLzYwDQpgYGANCg0K
