# Supplementary material for: Is the cholesterol-perfluoroalkyl substance association confounded by dietary fiber intake?: a Bayesian analysis of NHANES data with adjustment for measurement error in fiber intake
Source: Environ Health. 2022 Nov 22;21:114. doi: 10.1186/s12940-022-00923-2 (PMC9682702; doi:10.1186/s12940-022-00923-2)
Supplement: Supplementary file 4 — Additional file 4. [file 12940_2022_923_MOESM4_ESM.zip › Supplemental_Code/pfas_fiber_ols.nb.html]

R Notebook


Code 

- Show All Code
- Hide All Code
- Download Rmd

# R Notebook

# Ordinary Least Squares Regression

First the OLS is run.

## Data setup

Load in data


```
setwd(this.path::here())
indf <- read.csv("data_7-21-21.csv", header = T)
```


Now update data as needed


```
#Center income to poverty variable on its mean
indf$indfmpir_c <- indf$indfmpir - 2.545291
#Find the mean of first and second total fiber samples
indf$crude_F <- (indf$adj_lneafib1 +indf$adj_lneafib2)/2
#Find the mean of the first and second soluble fiber samples
indf$crude_Fsol <- (indf$adj_lneafsol1+indf$adj_lneafsol2)/2
```

## Ordinary Least Squares Analysis

Now we run the analysis for each chemical (PFOS, PFOA, PFNA) and with
each fiber scenario (no fiber, total fiber, soluble fiber)

### PFOA No Fiber


```
modAfit <- lm(lnchol ~  
            adj_pfoa + 
            adj_energy +
            adj_easatfat +
            adj_eadchol + 
            adj_age +
            sex +
            et1 + 
            et2 +
            et4 + 
            et5 +
            indfmpir_c +
            adj_bmi +
            smoker +
            wave +
            wave2 
          ,data = indf)

print(paste0("n = ", nrow(indf)))
```


```
[1] "n = 7242"
```


```
summary(modAfit)
```


```
Call:
lm(formula = lnchol ~ adj_pfoa + adj_energy + adj_easatfat + 
    adj_eadchol + adj_age + sex + et1 + et2 + et4 + et5 + indfmpir_c + 
    adj_bmi + smoker + wave + wave2, data = indf)

Residuals:
     Min       1Q   Median       3Q      Max 
-0.63285 -0.12373  0.00269  0.12496  0.58713 

Coefficients:
               Estimate Std. Error t value Pr(>|t|)    
(Intercept)   5.276e+00  6.983e-03 755.623  < 2e-16 ***
adj_pfoa      2.593e-03  7.452e-04   3.479 0.000505 ***
adj_energy   -3.767e-07  3.105e-06  -0.121 0.903439    
adj_easatfat  6.227e-05  3.171e-04   0.196 0.844300    
adj_eadchol   6.113e-06  1.482e-05   0.412 0.680005    
adj_age       2.889e-03  1.357e-04  21.280  < 2e-16 ***
sex           2.673e-02  4.961e-03   5.388 7.34e-08 ***
et1           1.782e-02  6.669e-03   2.673 0.007541 ** 
et2           2.845e-02  8.270e-03   3.440 0.000585 ***
et4          -2.820e-02  6.036e-03  -4.672 3.03e-06 ***
et5           5.581e-03  8.787e-03   0.635 0.525330    
indfmpir_c    6.433e-03  1.484e-03   4.335 1.48e-05 ***
adj_bmi       1.683e-03  3.258e-04   5.166 2.46e-07 ***
smoker        9.203e-03  2.691e-03   3.420 0.000629 ***
wave         -5.735e-03  4.029e-03  -1.424 0.154616    
wave2        -1.826e-04  6.451e-04  -0.283 0.777116    
---
Signif. codes:  0 ‘***’ 0.001 ‘**’ 0.01 ‘*’ 0.05 ‘.’ 0.1 ‘ ’ 1

Residual standard error: 0.1875 on 7226 degrees of freedom
Multiple R-squared:  0.09325,   Adjusted R-squared:  0.09137 
F-statistic: 49.54 on 15 and 7226 DF,  p-value: < 2.2e-16
```

### PFOA Total Fiber


```
modAfit <- lm(lnchol ~  
            adj_pfoa + 
            adj_energy +
            adj_easatfat +
            adj_eadchol + 
            adj_age +
            sex +
            et1 + 
            et2 +
            et4 + 
            et5 +
            indfmpir_c +
            adj_bmi +
            smoker +
            wave +
            wave2 + 
            crude_F
          ,data = indf)

print(paste0("n = ", nrow(indf)))
```


```
[1] "n = 7242"
```


```
summary(modAfit)
```


```
Call:
lm(formula = lnchol ~ adj_pfoa + adj_energy + adj_easatfat + 
    adj_eadchol + adj_age + sex + et1 + et2 + et4 + et5 + indfmpir_c + 
    adj_bmi + smoker + wave + wave2 + crude_F, data = indf)

Residuals:
     Min       1Q   Median       3Q      Max 
-0.63393 -0.12336  0.00316  0.12554  0.58551 

Coefficients:
               Estimate Std. Error t value Pr(>|t|)    
(Intercept)   5.276e+00  6.986e-03 755.206  < 2e-16 ***
adj_pfoa      2.530e-03  7.469e-04   3.387  0.00071 ***
adj_energy   -3.236e-07  3.105e-06  -0.104  0.91700    
adj_easatfat -3.896e-06  3.217e-04  -0.012  0.99034    
adj_eadchol   3.536e-06  1.497e-05   0.236  0.81331    
adj_age       2.924e-03  1.389e-04  21.058  < 2e-16 ***
sex           2.702e-02  4.966e-03   5.440 5.50e-08 ***
et1           1.925e-02  6.771e-03   2.843  0.00448 ** 
et2           2.859e-02  8.271e-03   3.457  0.00055 ***
et4          -2.909e-02  6.080e-03  -4.785 1.75e-06 ***
et5           5.979e-03  8.792e-03   0.680  0.49652    
indfmpir_c    6.606e-03  1.491e-03   4.432 9.48e-06 ***
adj_bmi       1.662e-03  3.263e-04   5.094 3.59e-07 ***
smoker        8.573e-03  2.740e-03   3.129  0.00176 ** 
wave         -5.471e-03  4.034e-03  -1.356  0.17509    
wave2        -2.092e-04  6.454e-04  -0.324  0.74581    
crude_F      -1.918e-02  1.578e-02  -1.216  0.22407    
---
Signif. codes:  0 ‘***’ 0.001 ‘**’ 0.01 ‘*’ 0.05 ‘.’ 0.1 ‘ ’ 1

Residual standard error: 0.1875 on 7225 degrees of freedom
Multiple R-squared:  0.09344,   Adjusted R-squared:  0.09143 
F-statistic: 46.54 on 16 and 7225 DF,  p-value: < 2.2e-16
```

### PFOA Soluble Fiber


```
modAfit <- lm(lnchol ~  
            adj_pfoa + 
            adj_energy +
            adj_easatfat +
            adj_eadchol + 
            adj_age +
            sex +
            et1 + 
            et2 +
            et4 + 
            et5 +
            indfmpir_c +
            adj_bmi +
            smoker +
            wave +
            wave2 +
            crude_Fsol
          ,data = indf)

print(paste0("n = ", nrow(indf)))
```


```
[1] "n = 7242"
```


```
summary(modAfit)
```


```
Call:
lm(formula = lnchol ~ adj_pfoa + adj_energy + adj_easatfat + 
    adj_eadchol + adj_age + sex + et1 + et2 + et4 + et5 + indfmpir_c + 
    adj_bmi + smoker + wave + wave2 + crude_Fsol, data = indf)

Residuals:
     Min       1Q   Median       3Q      Max 
-0.63513 -0.12427  0.00368  0.12558  0.58474 

Coefficients:
               Estimate Std. Error t value Pr(>|t|)    
(Intercept)   5.276e+00  6.981e-03 755.733  < 2e-16 ***
adj_pfoa      2.448e-03  7.467e-04   3.278 0.001049 ** 
adj_energy   -6.445e-07  3.105e-06  -0.208 0.835584    
adj_easatfat -8.408e-07  3.178e-04  -0.003 0.997889    
adj_eadchol   4.871e-07  1.496e-05   0.033 0.974019    
adj_age       2.945e-03  1.372e-04  21.459  < 2e-16 ***
sex           2.705e-02  4.960e-03   5.454 5.08e-08 ***
et1           2.119e-02  6.779e-03   3.126 0.001779 ** 
et2           2.843e-02  8.266e-03   3.439 0.000588 ***
et4          -3.026e-02  6.080e-03  -4.977 6.61e-07 ***
et5           5.202e-03  8.784e-03   0.592 0.553696    
indfmpir_c    6.612e-03  1.485e-03   4.454 8.57e-06 ***
adj_bmi       1.653e-03  3.258e-04   5.074 3.99e-07 ***
smoker        8.027e-03  2.724e-03   2.947 0.003219 ** 
wave         -5.217e-03  4.031e-03  -1.294 0.195665    
wave2        -2.335e-04  6.450e-04  -0.362 0.717405    
crude_Fsol   -2.900e-02  1.062e-02  -2.731 0.006328 ** 
---
Signif. codes:  0 ‘***’ 0.001 ‘**’ 0.01 ‘*’ 0.05 ‘.’ 0.1 ‘ ’ 1

Residual standard error: 0.1874 on 7225 degrees of freedom
Multiple R-squared:  0.09419,   Adjusted R-squared:  0.09218 
F-statistic: 46.95 on 16 and 7225 DF,  p-value: < 2.2e-16
```

### PFOS No Fiber


```
modAfit <- lm(lnchol ~  
            adj_pfos + 
            adj_energy +
            adj_easatfat +
            adj_eadchol + 
            adj_age +
            sex +
            et1 + 
            et2 +
            et4 + 
            et5 +
            indfmpir_c +
            adj_bmi +
            smoker +
            wave +
            wave2
          ,data = indf)

print(paste0("n = ", nrow(indf)))
```


```
[1] "n = 7242"
```


```
summary(modAfit)
```


```
Call:
lm(formula = lnchol ~ adj_pfos + adj_energy + adj_easatfat + 
    adj_eadchol + adj_age + sex + et1 + et2 + et4 + et5 + indfmpir_c + 
    adj_bmi + smoker + wave + wave2, data = indf)

Residuals:
     Min       1Q   Median       3Q      Max 
-0.63121 -0.12374  0.00382  0.12558  0.58810 

Coefficients:
               Estimate Std. Error t value Pr(>|t|)    
(Intercept)   5.269e+00  7.220e-03 729.756  < 2e-16 ***
adj_pfos      8.399e-04  1.619e-04   5.188 2.18e-07 ***
adj_energy    2.397e-07  3.104e-06   0.077 0.938437    
adj_easatfat  1.033e-04  3.169e-04   0.326 0.744510    
adj_eadchol   3.870e-06  1.482e-05   0.261 0.793952    
adj_age       2.785e-03  1.380e-04  20.186  < 2e-16 ***
sex           2.925e-02  4.998e-03   5.853 5.05e-09 ***
et1           1.829e-02  6.653e-03   2.749 0.005984 ** 
et2           2.936e-02  8.265e-03   3.553 0.000383 ***
et4          -3.214e-02  6.070e-03  -5.294 1.23e-07 ***
et5           3.099e-03  8.780e-03   0.353 0.724124    
indfmpir_c    6.573e-03  1.478e-03   4.446 8.89e-06 ***
adj_bmi       1.712e-03  3.256e-04   5.260 1.48e-07 ***
smoker        9.826e-03  2.688e-03   3.656 0.000258 ***
wave         -1.953e-03  4.101e-03  -0.476 0.633967    
wave2        -5.707e-04  6.446e-04  -0.885 0.376051    
---
Signif. codes:  0 ‘***’ 0.001 ‘**’ 0.01 ‘*’ 0.05 ‘.’ 0.1 ‘ ’ 1

Residual standard error: 0.1873 on 7226 degrees of freedom
Multiple R-squared:  0.09511,   Adjusted R-squared:  0.09323 
F-statistic: 50.63 on 15 and 7226 DF,  p-value: < 2.2e-16
```

### PFOS Total Fiber


```
modAfit <- lm(lnchol ~  
            adj_pfos + 
            adj_energy +
            adj_easatfat +
            adj_eadchol + 
            adj_age +
            sex +
            et1 + 
            et2 +
            et4 + 
            et5 +
            indfmpir_c +
            adj_bmi +
            smoker +
            wave +
            wave2 +
            crude_F
          ,data = indf)

print(paste0("n = ", nrow(indf)))
```


```
[1] "n = 7242"
```


```
summary(modAfit)
```


```
Call:
lm(formula = lnchol ~ adj_pfos + adj_energy + adj_easatfat + 
    adj_eadchol + adj_age + sex + et1 + et2 + et4 + et5 + indfmpir_c + 
    adj_bmi + smoker + wave + wave2 + crude_F, data = indf)

Residuals:
     Min       1Q   Median       3Q      Max 
-0.63222 -0.12431  0.00425  0.12568  0.58661 

Coefficients:
               Estimate Std. Error t value Pr(>|t|)    
(Intercept)   5.269e+00  7.222e-03 729.533  < 2e-16 ***
adj_pfos      8.283e-04  1.622e-04   5.106 3.37e-07 ***
adj_energy    2.800e-07  3.104e-06   0.090 0.928132    
adj_easatfat  4.154e-05  3.216e-04   0.129 0.897221    
adj_eadchol   1.502e-06  1.497e-05   0.100 0.920067    
adj_age       2.819e-03  1.412e-04  19.961  < 2e-16 ***
sex           2.950e-02  5.003e-03   5.897 3.87e-09 ***
et1           1.963e-02  6.757e-03   2.904 0.003691 ** 
et2           2.949e-02  8.265e-03   3.568 0.000362 ***
et4          -3.290e-02  6.108e-03  -5.387 7.40e-08 ***
et5           3.509e-03  8.788e-03   0.399 0.689699    
indfmpir_c    6.728e-03  1.485e-03   4.531 5.96e-06 ***
adj_bmi       1.693e-03  3.260e-04   5.192 2.14e-07 ***
smoker        9.232e-03  2.739e-03   3.371 0.000753 ***
wave         -1.758e-03  4.104e-03  -0.428 0.668368    
wave2        -5.886e-04  6.448e-04  -0.913 0.361338    
crude_F      -1.775e-02  1.576e-02  -1.127 0.259990    
---
Signif. codes:  0 ‘***’ 0.001 ‘**’ 0.01 ‘*’ 0.05 ‘.’ 0.1 ‘ ’ 1

Residual standard error: 0.1873 on 7225 degrees of freedom
Multiple R-squared:  0.09526,   Adjusted R-squared:  0.09326 
F-statistic: 47.55 on 16 and 7225 DF,  p-value: < 2.2e-16
```

### PFOS Soluble Fiber


```
modAfit <- lm(lnchol ~  
            adj_pfos + 
            adj_energy +
            adj_easatfat +
            adj_eadchol + 
            adj_age +
            sex +
            et1 + 
            et2 +
            et4 + 
            et5 +
            indfmpir_c +
            adj_bmi +
            smoker +
            wave +
            wave2 +
            crude_Fsol
          ,data = indf)

print(paste0("n = ", nrow(indf)))
```


```
[1] "n = 7242"
```


```
summary(modAfit)
```


```
Call:
lm(formula = lnchol ~ adj_pfos + adj_energy + adj_easatfat + 
    adj_eadchol + adj_age + sex + et1 + et2 + et4 + et5 + indfmpir_c + 
    adj_bmi + smoker + wave + wave2 + crude_Fsol, data = indf)

Residuals:
    Min      1Q  Median      3Q     Max 
-0.6334 -0.1239  0.0042  0.1254  0.5858 

Coefficients:
               Estimate Std. Error t value Pr(>|t|)    
(Intercept)   5.269e+00  7.218e-03 729.977  < 2e-16 ***
adj_pfos      8.099e-04  1.622e-04   4.992 6.11e-07 ***
adj_energy   -3.896e-08  3.105e-06  -0.013 0.989988    
adj_easatfat  4.161e-05  3.176e-04   0.131 0.895775    
adj_eadchol  -1.457e-06  1.495e-05  -0.097 0.922384    
adj_age       2.842e-03  1.396e-04  20.356  < 2e-16 ***
sex           2.951e-02  4.997e-03   5.906 3.67e-09 ***
et1           2.153e-02  6.765e-03   3.183 0.001465 ** 
et2           2.933e-02  8.261e-03   3.550 0.000388 ***
et4          -3.396e-02  6.108e-03  -5.560 2.79e-08 ***
et5           2.840e-03  8.777e-03   0.324 0.746241    
indfmpir_c    6.732e-03  1.479e-03   4.551 5.42e-06 ***
adj_bmi       1.683e-03  3.256e-04   5.170 2.41e-07 ***
smoker        8.675e-03  2.722e-03   3.187 0.001445 ** 
wave         -1.587e-03  4.101e-03  -0.387 0.698787    
wave2        -6.028e-04  6.445e-04  -0.935 0.349688    
crude_Fsol   -2.771e-02  1.061e-02  -2.613 0.009001 ** 
---
Signif. codes:  0 ‘***’ 0.001 ‘**’ 0.01 ‘*’ 0.05 ‘.’ 0.1 ‘ ’ 1

Residual standard error: 0.1872 on 7225 degrees of freedom
Multiple R-squared:  0.09596,   Adjusted R-squared:  0.09396 
F-statistic: 47.93 on 16 and 7225 DF,  p-value: < 2.2e-16
```

### PFNA No Fiber


```
modAfit <- lm(lnchol ~  
            adj_pfna + 
            adj_energy +
            adj_easatfat +
            adj_eadchol + 
            adj_age +
            sex +
            et1 + 
            et2 +
            et4 + 
            et5 +
            indfmpir_c +
            adj_bmi +
            smoker + 
            wave +
            wave2 
          ,data = indf)

print(paste0("n = ", nrow(indf)))
```


```
[1] "n = 7242"
```


```
summary(modAfit)
```


```
Call:
lm(formula = lnchol ~ adj_pfna + adj_energy + adj_easatfat + 
    adj_eadchol + adj_age + sex + et1 + et2 + et4 + et5 + indfmpir_c + 
    adj_bmi + smoker + wave + wave2, data = indf)

Residuals:
     Min       1Q   Median       3Q      Max 
-0.62929 -0.12404  0.00305  0.12517  0.58234 

Coefficients:
               Estimate Std. Error t value Pr(>|t|)    
(Intercept)   5.280e+00  6.860e-03 769.721  < 2e-16 ***
adj_pfna      8.354e-03  1.976e-03   4.227 2.40e-05 ***
adj_energy   -1.238e-07  3.104e-06  -0.040  0.96820    
adj_easatfat  1.083e-04  3.172e-04   0.342  0.73273    
adj_eadchol   3.681e-06  1.484e-05   0.248  0.80410    
adj_age       2.881e-03  1.357e-04  21.238  < 2e-16 ***
sex           2.622e-02  4.931e-03   5.317 1.09e-07 ***
et1           1.721e-02  6.648e-03   2.589  0.00965 ** 
et2           2.692e-02  8.263e-03   3.258  0.00113 ** 
et4          -3.058e-02  6.053e-03  -5.053 4.47e-07 ***
et5           3.010e-03  8.790e-03   0.342  0.73200    
indfmpir_c    6.489e-03  1.481e-03   4.382 1.19e-05 ***
adj_bmi       1.671e-03  3.256e-04   5.133 2.93e-07 ***
smoker        9.377e-03  2.689e-03   3.488  0.00049 ***
wave         -7.633e-03  4.043e-03  -1.888  0.05908 .  
wave2         7.256e-05  6.504e-04   0.112  0.91118    
---
Signif. codes:  0 ‘***’ 0.001 ‘**’ 0.01 ‘*’ 0.05 ‘.’ 0.1 ‘ ’ 1

Residual standard error: 0.1874 on 7226 degrees of freedom
Multiple R-squared:  0.09397,   Adjusted R-squared:  0.09209 
F-statistic: 49.97 on 15 and 7226 DF,  p-value: < 2.2e-16
```

### PFNA Total Fiber


```
modAfit <- lm(lnchol ~  
            adj_pfna + 
            adj_energy +
            adj_easatfat +
            adj_eadchol + 
            adj_age +
            sex +
            et1 + 
            et2 +
            et4 + 
            et5 +
            indfmpir_c +
            adj_bmi +
            smoker +
            wave +
            wave2 +
            crude_F
          ,data = indf)

print(paste0("n = ", nrow(indf)))
```


```
[1] "n = 7242"
```


```
summary(modAfit)
```


```
Call:
lm(formula = lnchol ~ adj_pfna + adj_energy + adj_easatfat + 
    adj_eadchol + adj_age + sex + et1 + et2 + et4 + et5 + indfmpir_c + 
    adj_bmi + smoker + wave + wave2 + crude_F, data = indf)

Residuals:
     Min       1Q   Median       3Q      Max 
-0.63037 -0.12412  0.00335  0.12529  0.58092 

Coefficients:
               Estimate Std. Error t value Pr(>|t|)    
(Intercept)   5.280e+00  6.866e-03 768.948  < 2e-16 ***
adj_pfna      8.182e-03  1.982e-03   4.128 3.70e-05 ***
adj_energy   -7.954e-08  3.105e-06  -0.026  0.97956    
adj_easatfat  4.570e-05  3.219e-04   0.142  0.88713    
adj_eadchol   1.325e-06  1.498e-05   0.088  0.92954    
adj_age       2.914e-03  1.388e-04  20.997  < 2e-16 ***
sex           2.650e-02  4.937e-03   5.367 8.25e-08 ***
et1           1.856e-02  6.753e-03   2.748  0.00601 ** 
et2           2.708e-02  8.264e-03   3.277  0.00105 ** 
et4          -3.136e-02  6.092e-03  -5.148 2.70e-07 ***
et5           3.436e-03  8.797e-03   0.391  0.69614    
indfmpir_c    6.648e-03  1.488e-03   4.469 7.97e-06 ***
adj_bmi       1.652e-03  3.261e-04   5.067 4.15e-07 ***
smoker        8.786e-03  2.739e-03   3.208  0.00134 ** 
wave         -7.347e-03  4.051e-03  -1.814  0.06976 .  
wave2         4.277e-05  6.510e-04   0.066  0.94761    
crude_F      -1.788e-02  1.578e-02  -1.133  0.25714    
---
Signif. codes:  0 ‘***’ 0.001 ‘**’ 0.01 ‘*’ 0.05 ‘.’ 0.1 ‘ ’ 1

Residual standard error: 0.1874 on 7225 degrees of freedom
Multiple R-squared:  0.09414,   Adjusted R-squared:  0.09213 
F-statistic: 46.93 on 16 and 7225 DF,  p-value: < 2.2e-16
```

### PFNA Soluble Fiber


```
modAfit <- lm(lnchol ~  
            adj_pfna + 
            adj_energy +
            adj_easatfat +
            adj_eadchol + 
            adj_age +
            sex +
            et1 + 
            et2 +
            et4 + 
            et5 +
            indfmpir_c +
            adj_bmi +
            smoker +
            wave +
            wave2 +
            crude_Fsol
          ,data = indf)

print(paste0("n = ", nrow(indf)))
```


```
[1] "n = 7242"
```


```
summary(modAfit)
```


```
Call:
lm(formula = lnchol ~ adj_pfna + adj_energy + adj_easatfat + 
    adj_eadchol + adj_age + sex + et1 + et2 + et4 + et5 + indfmpir_c + 
    adj_bmi + smoker + wave + wave2 + crude_Fsol, data = indf)

Residuals:
     Min       1Q   Median       3Q      Max 
-0.63166 -0.12439  0.00385  0.12579  0.58032 

Coefficients:
               Estimate Std. Error t value Pr(>|t|)    
(Intercept)   5.279e+00  6.860e-03 769.548  < 2e-16 ***
adj_pfna      7.883e-03  1.984e-03   3.974 7.15e-05 ***
adj_energy   -3.928e-07  3.105e-06  -0.127  0.89933    
adj_easatfat  4.562e-05  3.180e-04   0.143  0.88592    
adj_eadchol  -1.532e-06  1.497e-05  -0.102  0.91848    
adj_age       2.935e-03  1.372e-04  21.394  < 2e-16 ***
sex           2.655e-02  4.931e-03   5.384 7.53e-08 ***
et1           2.044e-02  6.761e-03   3.024  0.00251 ** 
et2           2.698e-02  8.259e-03   3.266  0.00109 ** 
et4          -3.241e-02  6.092e-03  -5.321 1.06e-07 ***
et5           2.792e-03  8.787e-03   0.318  0.75068    
indfmpir_c    6.657e-03  1.482e-03   4.493 7.14e-06 ***
adj_bmi       1.644e-03  3.257e-04   5.047 4.59e-07 ***
smoker        8.249e-03  2.722e-03   3.030  0.00245 ** 
wave         -7.034e-03  4.048e-03  -1.738  0.08233 .  
wave2         9.273e-06  6.506e-04   0.014  0.98863    
crude_Fsol   -2.761e-02  1.063e-02  -2.596  0.00944 ** 
---
Signif. codes:  0 ‘***’ 0.001 ‘**’ 0.01 ‘*’ 0.05 ‘.’ 0.1 ‘ ’ 1

Residual standard error: 0.1873 on 7225 degrees of freedom
Multiple R-squared:  0.09482,   Adjusted R-squared:  0.09281 
F-statistic:  47.3 on 16 and 7225 DF,  p-value: < 2.2e-16
```

LS0tDQp0aXRsZTogIlIgTm90ZWJvb2siDQpvdXRwdXQ6IGh0bWxfbm90ZWJvb2sNCi0tLQ0KDQojIE9yZGluYXJ5IExlYXN0IFNxdWFyZXMgUmVncmVzc2lvbg0KDQpGaXJzdCB0aGUgT0xTIGlzIHJ1bi4NCg0KIyMgRGF0YSBzZXR1cA0KDQpMb2FkIGluIGRhdGENCmBgYHtyfQ0Kc2V0d2QodGhpcy5wYXRoOjpoZXJlKCkpDQppbmRmIDwtIHJlYWQuY3N2KCJkYXRhXzctMjEtMjEuY3N2IiwgaGVhZGVyID0gVCkNCmBgYA0KDQpOb3cgdXBkYXRlIGRhdGEgYXMgbmVlZGVkDQpgYGB7cn0NCiNDZW50ZXIgaW5jb21lIHRvIHBvdmVydHkgdmFyaWFibGUgb24gaXRzIG1lYW4NCmluZGYkaW5kZm1waXJfYyA8LSBpbmRmJGluZGZtcGlyIC0gMi41NDUyOTENCiNGaW5kIHRoZSBtZWFuIG9mIGZpcnN0IGFuZCBzZWNvbmQgdG90YWwgZmliZXIgc2FtcGxlcw0KaW5kZiRjcnVkZV9GIDwtIChpbmRmJGFkal9sbmVhZmliMSAraW5kZiRhZGpfbG5lYWZpYjIpLzINCiNGaW5kIHRoZSBtZWFuIG9mIHRoZSBmaXJzdCBhbmQgc2Vjb25kIHNvbHVibGUgZmliZXIgc2FtcGxlcw0KaW5kZiRjcnVkZV9Gc29sIDwtIChpbmRmJGFkal9sbmVhZnNvbDEraW5kZiRhZGpfbG5lYWZzb2wyKS8yDQpgYGANCg0KIyMgT3JkaW5hcnkgTGVhc3QgU3F1YXJlcyBBbmFseXNpcw0KDQpOb3cgd2UgcnVuIHRoZSBhbmFseXNpcyBmb3IgZWFjaCBjaGVtaWNhbCAoUEZPUywgUEZPQSwgUEZOQSkgYW5kIHdpdGggZWFjaCBmaWJlciBzY2VuYXJpbyAobm8gZmliZXIsIHRvdGFsIGZpYmVyLCBzb2x1YmxlIGZpYmVyKQ0KDQojIyMgUEZPQSBObyBGaWJlcg0KDQpgYGB7cn0NCm1vZEFmaXQgPC0gbG0obG5jaG9sIH4gIA0KICAgICAgICAgICAgYWRqX3Bmb2EgKyANCiAgICAgICAgICAgIGFkal9lbmVyZ3kgKw0KICAgICAgICAgICAgYWRqX2Vhc2F0ZmF0ICsNCiAgICAgICAgICAgIGFkal9lYWRjaG9sICsgDQogICAgICAgICAgICBhZGpfYWdlICsNCiAgICAgICAgICAgIHNleCArDQogICAgICAgICAgICBldDEgKyANCiAgICAgICAgICAgIGV0MiArDQogICAgICAgICAgICBldDQgKyANCiAgICAgICAgICAgIGV0NSArDQogICAgICAgICAgICBpbmRmbXBpcl9jICsNCiAgICAgICAgICAgIGFkal9ibWkgKw0KICAgICAgICAgICAgc21va2VyICsNCiAgICAgICAgICAgIHdhdmUgKw0KICAgICAgICAgICAgd2F2ZTIgDQogICAgICAgICAgLGRhdGEgPSBpbmRmKQ0KDQpwcmludChwYXN0ZTAoIm4gPSAiLCBucm93KGluZGYpKSkNCnN1bW1hcnkobW9kQWZpdCkNCmBgYA0KDQojIyMgUEZPQSBUb3RhbCBGaWJlcg0KDQpgYGB7cn0NCm1vZEFmaXQgPC0gbG0obG5jaG9sIH4gIA0KICAgICAgICAgICAgYWRqX3Bmb2EgKyANCiAgICAgICAgICAgIGFkal9lbmVyZ3kgKw0KICAgICAgICAgICAgYWRqX2Vhc2F0ZmF0ICsNCiAgICAgICAgICAgIGFkal9lYWRjaG9sICsgDQogICAgICAgICAgICBhZGpfYWdlICsNCiAgICAgICAgICAgIHNleCArDQogICAgICAgICAgICBldDEgKyANCiAgICAgICAgICAgIGV0MiArDQogICAgICAgICAgICBldDQgKyANCiAgICAgICAgICAgIGV0NSArDQogICAgICAgICAgICBpbmRmbXBpcl9jICsNCiAgICAgICAgICAgIGFkal9ibWkgKw0KICAgICAgICAgICAgc21va2VyICsNCiAgICAgICAgICAgIHdhdmUgKw0KICAgICAgICAgICAgd2F2ZTIgKyANCiAgICAgICAgICAgIGNydWRlX0YNCiAgICAgICAgICAsZGF0YSA9IGluZGYpDQoNCnByaW50KHBhc3RlMCgibiA9ICIsIG5yb3coaW5kZikpKQ0Kc3VtbWFyeShtb2RBZml0KQ0KYGBgDQoNCiMjIyBQRk9BIFNvbHVibGUgRmliZXINCg0KYGBge3J9DQptb2RBZml0IDwtIGxtKGxuY2hvbCB+ICANCiAgICAgICAgICAgIGFkal9wZm9hICsgDQogICAgICAgICAgICBhZGpfZW5lcmd5ICsNCiAgICAgICAgICAgIGFkal9lYXNhdGZhdCArDQogICAgICAgICAgICBhZGpfZWFkY2hvbCArIA0KICAgICAgICAgICAgYWRqX2FnZSArDQogICAgICAgICAgICBzZXggKw0KICAgICAgICAgICAgZXQxICsgDQogICAgICAgICAgICBldDIgKw0KICAgICAgICAgICAgZXQ0ICsgDQogICAgICAgICAgICBldDUgKw0KICAgICAgICAgICAgaW5kZm1waXJfYyArDQogICAgICAgICAgICBhZGpfYm1pICsNCiAgICAgICAgICAgIHNtb2tlciArDQogICAgICAgICAgICB3YXZlICsNCiAgICAgICAgICAgIHdhdmUyICsNCiAgICAgICAgICAgIGNydWRlX0Zzb2wNCiAgICAgICAgICAsZGF0YSA9IGluZGYpDQoNCnByaW50KHBhc3RlMCgibiA9ICIsIG5yb3coaW5kZikpKQ0Kc3VtbWFyeShtb2RBZml0KQ0KYGBgDQoNCiMjIyBQRk9TIE5vIEZpYmVyDQoNCmBgYHtyfQ0KbW9kQWZpdCA8LSBsbShsbmNob2wgfiAgDQogICAgICAgICAgICBhZGpfcGZvcyArIA0KICAgICAgICAgICAgYWRqX2VuZXJneSArDQogICAgICAgICAgICBhZGpfZWFzYXRmYXQgKw0KICAgICAgICAgICAgYWRqX2VhZGNob2wgKyANCiAgICAgICAgICAgIGFkal9hZ2UgKw0KICAgICAgICAgICAgc2V4ICsNCiAgICAgICAgICAgIGV0MSArIA0KICAgICAgICAgICAgZXQyICsNCiAgICAgICAgICAgIGV0NCArIA0KICAgICAgICAgICAgZXQ1ICsNCiAgICAgICAgICAgIGluZGZtcGlyX2MgKw0KICAgICAgICAgICAgYWRqX2JtaSArDQogICAgICAgICAgICBzbW9rZXIgKw0KICAgICAgICAgICAgd2F2ZSArDQogICAgICAgICAgICB3YXZlMg0KICAgICAgICAgICxkYXRhID0gaW5kZikNCg0KcHJpbnQocGFzdGUwKCJuID0gIiwgbnJvdyhpbmRmKSkpDQpzdW1tYXJ5KG1vZEFmaXQpDQpgYGANCg0KIyMjIFBGT1MgVG90YWwgRmliZXINCg0KYGBge3J9DQptb2RBZml0IDwtIGxtKGxuY2hvbCB+ICANCiAgICAgICAgICAgIGFkal9wZm9zICsgDQogICAgICAgICAgICBhZGpfZW5lcmd5ICsNCiAgICAgICAgICAgIGFkal9lYXNhdGZhdCArDQogICAgICAgICAgICBhZGpfZWFkY2hvbCArIA0KICAgICAgICAgICAgYWRqX2FnZSArDQogICAgICAgICAgICBzZXggKw0KICAgICAgICAgICAgZXQxICsgDQogICAgICAgICAgICBldDIgKw0KICAgICAgICAgICAgZXQ0ICsgDQogICAgICAgICAgICBldDUgKw0KICAgICAgICAgICAgaW5kZm1waXJfYyArDQogICAgICAgICAgICBhZGpfYm1pICsNCiAgICAgICAgICAgIHNtb2tlciArDQogICAgICAgICAgICB3YXZlICsNCiAgICAgICAgICAgIHdhdmUyICsNCiAgICAgICAgICAgIGNydWRlX0YNCiAgICAgICAgICAsZGF0YSA9IGluZGYpDQoNCnByaW50KHBhc3RlMCgibiA9ICIsIG5yb3coaW5kZikpKQ0Kc3VtbWFyeShtb2RBZml0KQ0KYGBgDQoNCiMjIyBQRk9TIFNvbHVibGUgRmliZXINCg0KYGBge3J9DQptb2RBZml0IDwtIGxtKGxuY2hvbCB+ICANCiAgICAgICAgICAgIGFkal9wZm9zICsgDQogICAgICAgICAgICBhZGpfZW5lcmd5ICsNCiAgICAgICAgICAgIGFkal9lYXNhdGZhdCArDQogICAgICAgICAgICBhZGpfZWFkY2hvbCArIA0KICAgICAgICAgICAgYWRqX2FnZSArDQogICAgICAgICAgICBzZXggKw0KICAgICAgICAgICAgZXQxICsgDQogICAgICAgICAgICBldDIgKw0KICAgICAgICAgICAgZXQ0ICsgDQogICAgICAgICAgICBldDUgKw0KICAgICAgICAgICAgaW5kZm1waXJfYyArDQogICAgICAgICAgICBhZGpfYm1pICsNCiAgICAgICAgICAgIHNtb2tlciArDQogICAgICAgICAgICB3YXZlICsNCiAgICAgICAgICAgIHdhdmUyICsNCiAgICAgICAgICAgIGNydWRlX0Zzb2wNCiAgICAgICAgICAsZGF0YSA9IGluZGYpDQoNCnByaW50KHBhc3RlMCgibiA9ICIsIG5yb3coaW5kZikpKQ0Kc3VtbWFyeShtb2RBZml0KQ0KYGBgDQoNCiMjIyBQRk5BIE5vIEZpYmVyDQoNCmBgYHtyfQ0KbW9kQWZpdCA8LSBsbShsbmNob2wgfiAgDQogICAgICAgICAgICBhZGpfcGZuYSArIA0KICAgICAgICAgICAgYWRqX2VuZXJneSArDQogICAgICAgICAgICBhZGpfZWFzYXRmYXQgKw0KICAgICAgICAgICAgYWRqX2VhZGNob2wgKyANCiAgICAgICAgICAgIGFkal9hZ2UgKw0KICAgICAgICAgICAgc2V4ICsNCiAgICAgICAgICAgIGV0MSArIA0KICAgICAgICAgICAgZXQyICsNCiAgICAgICAgICAgIGV0NCArIA0KICAgICAgICAgICAgZXQ1ICsNCiAgICAgICAgICAgIGluZGZtcGlyX2MgKw0KICAgICAgICAgICAgYWRqX2JtaSArDQogICAgICAgICAgICBzbW9rZXIgKyANCiAgICAgICAgICAgIHdhdmUgKw0KICAgICAgICAgICAgd2F2ZTIgDQogICAgICAgICAgLGRhdGEgPSBpbmRmKQ0KDQpwcmludChwYXN0ZTAoIm4gPSAiLCBucm93KGluZGYpKSkNCnN1bW1hcnkobW9kQWZpdCkNCmBgYA0KDQojIyMgUEZOQSBUb3RhbCBGaWJlcg0KDQpgYGB7cn0NCm1vZEFmaXQgPC0gbG0obG5jaG9sIH4gIA0KICAgICAgICAgICAgYWRqX3BmbmEgKyANCiAgICAgICAgICAgIGFkal9lbmVyZ3kgKw0KICAgICAgICAgICAgYWRqX2Vhc2F0ZmF0ICsNCiAgICAgICAgICAgIGFkal9lYWRjaG9sICsgDQogICAgICAgICAgICBhZGpfYWdlICsNCiAgICAgICAgICAgIHNleCArDQogICAgICAgICAgICBldDEgKyANCiAgICAgICAgICAgIGV0MiArDQogICAgICAgICAgICBldDQgKyANCiAgICAgICAgICAgIGV0NSArDQogICAgICAgICAgICBpbmRmbXBpcl9jICsNCiAgICAgICAgICAgIGFkal9ibWkgKw0KICAgICAgICAgICAgc21va2VyICsNCiAgICAgICAgICAgIHdhdmUgKw0KICAgICAgICAgICAgd2F2ZTIgKw0KICAgICAgICAgICAgY3J1ZGVfRg0KICAgICAgICAgICxkYXRhID0gaW5kZikNCg0KcHJpbnQocGFzdGUwKCJuID0gIiwgbnJvdyhpbmRmKSkpDQpzdW1tYXJ5KG1vZEFmaXQpDQpgYGANCg0KIyMjIFBGTkEgU29sdWJsZSBGaWJlcg0KDQpgYGB7cn0NCm1vZEFmaXQgPC0gbG0obG5jaG9sIH4gIA0KICAgICAgICAgICAgYWRqX3BmbmEgKyANCiAgICAgICAgICAgIGFkal9lbmVyZ3kgKw0KICAgICAgICAgICAgYWRqX2Vhc2F0ZmF0ICsNCiAgICAgICAgICAgIGFkal9lYWRjaG9sICsgDQogICAgICAgICAgICBhZGpfYWdlICsNCiAgICAgICAgICAgIHNleCArDQogICAgICAgICAgICBldDEgKyANCiAgICAgICAgICAgIGV0MiArDQogICAgICAgICAgICBldDQgKyANCiAgICAgICAgICAgIGV0NSArDQogICAgICAgICAgICBpbmRmbXBpcl9jICsNCiAgICAgICAgICAgIGFkal9ibWkgKw0KICAgICAgICAgICAgc21va2VyICsNCiAgICAgICAgICAgIHdhdmUgKw0KICAgICAgICAgICAgd2F2ZTIgKw0KICAgICAgICAgICAgY3J1ZGVfRnNvbA0KICAgICAgICAgICxkYXRhID0gaW5kZikNCg0KcHJpbnQocGFzdGUwKCJuID0gIiwgbnJvdyhpbmRmKSkpDQpzdW1tYXJ5KG1vZEFmaXQpDQpgYGANCg==
